# Single-Cell Growth Rates in Photoautotrophic Populations Measured by Stable Isotope Probing and Resonance Raman Microspectrometry

**DOI:** 10.3389/fmicb.2017.01449

**Published:** 2017-08-03

**Authors:** Gordon T. Taylor, Elizabeth A. Suter, Zhuo Q. Li, Stephanie Chow, Dallyce Stinton, Tatiana Zaliznyak, Steven R. Beaupré

**Affiliations:** School of Marine and Atmospheric Sciences, Stony Brook University Stony Brook, NY, United States

**Keywords:** single-cell analysis, carbon fixation, photosynthesis, productivity, intraspecific trait variability, Raman microspectrometry, carotenoids, stable isotope probing

## Abstract

A new method to measure growth rates of individual photoautotrophic cells by combining stable isotope probing (SIP) and single-cell resonance Raman microspectrometry is introduced. This report explores optimal experimental design and the theoretical underpinnings for quantitative responses of Raman spectra to cellular isotopic composition. Resonance Raman spectra of isogenic cultures of the cyanobacterium, *Synechococcus* sp., grown in ^13^C-bicarbonate revealed linear covariance between wavenumber (cm^−1^) shifts in dominant carotenoid Raman peaks and a broad range of cellular ^13^C fractional isotopic abundance. Single-cell growth rates were calculated from spectra-derived isotopic content and empirical relationships. Growth rates among any 25 cells in a sample varied considerably; mean coefficient of variation, CV, was 29 ± 3% (σ/$\bar{x}$), of which only ~2% was propagated analytical error. Instantaneous population growth rates measured independently by *in vivo* fluorescence also varied daily (CV ≈ 53%) and were statistically indistinguishable from single-cell growth rates at all but the lowest levels of cell labeling. SCRR censuses of mixtures prepared from *Synechococcus* sp. and *T. pseudonana* (a diatom) populations with varying ^13^C-content and growth rates closely approximated predicted spectral responses and fractional labeling of cells added to the sample. This approach enables direct microspectrometric interrogation of isotopically- and phylogenetically-labeled cells and detects as little as 3% changes in cellular fractional labeling. This is the first description of a non-destructive technique to measure single-cell photoautotrophic growth rates based on Raman spectroscopy and well-constrained assumptions, while requiring few ancillary measurements.

## Introduction

Ecological theory suggests that predictions about population dynamics in planktonic systems have limited skill if intraspecific trait variation and heterogeneous resource distributions are ignored. Intraspecific trait variation within microalgal and bacterial populations has been shown to non-linearly affect intra- and interspecific interactions and population responses to resource availability, pathogens, and predators (Hellweger and Kianirad, 2007; Bolnick et al., 2011; Bucci et al., 2012). Such trait variations can arise from any of the following: physiological history of individual cell lines, niche plasticity, natural selection, mutation, genetic drift, or recombinant events (Bolnick et al., 2011). Even clonal populations (isogenic) in apparently homogenous cultures exhibit varying genetics, biochemistry, physiology, and behavior, all of which can produce a range of “growth phenotypes” (Lidstrom and Konopka, 2010; Damodaran et al., 2015; Kopf et al., 2015). This recognition has given rise to agent-based or individual-based models which don't assume that cell attributes within a population are uniformly or normally distributed around their mean values (e.g., Hellweger and Kianirad, 2007). In addition, aquatic ecologists and biogeochemists now recognize that the planktonic realm has fine-scale structure imposed by heterogeneous distributions of nutrients, oxygen, particles, colonial microbes, and polymeric gels, as well as by metazoan behavior, and symbiotic associations (Azam, 1998; Simon et al., 2002; Wagner et al., 2006; Stocker, 2012; Gemmell et al., 2016). Historically however, the vast majority of measurements have overlooked this microspatial heterogeneity and cryptic material exchanges (e.g., Canfield et al., 2010), and have provided net biogeochemical transformations at best. They also fail to unequivocally link key players to particular processes. These limitations hamper a deeper mechanistic understanding of planktonic dynamics. Single-cell techniques are the only way forward to empirically determine how intraspecific phenotypic/genotypic variations and microspatial architecture (the aquascape) determine individual ecophysiologies and translate into collective population responses.

Several single-cell technologies have enabled examination of intra-population variability in genotype, phenotype, activity and cellular growth. Single-cell genomics and transcriptomics provide unique information on genetic variations and are gaining popularity in the aquatic sciences (Stepanauskas, 2012; Wu et al., 2014). The synchrotron X-ray microprobe has provided insights into intraspecific variability in individual cell elemental stoichiometries (Twining et al., 2008). Highly sensitive serial resonant mass sensor arrays have enabled measurement of changes in buoyant masses of bacterial and mammalian cells as they transit through microfluidic channels, thereby tracking somatic growth of individual cells through time (Cermak et al., 2016). Combining fluorescent *in situ* hybridization (FISH) with microautoradiography (MAR-FISH) (Lee et al., 1999) or with secondary ion mass spectrometry (nano-SIMS-FISH) (Orphan et al., 2002) provides single-cell resolution for linking identity to ecophysiology in complex microbial assemblages. Nano-SIMS-FISH has been combined with SIP to detect nutrient assimilation by individual cells (Musat et al., 2008; Orphan et al., 2009; Foster et al., 2011). Using deuterated water (D_2_O) and ^15^${\text{NH}}_{4}^{+}$ as tracers and SIP-Nano-SIMS analysis enabled Kopf et al. (2015) to demonstrate that intra-population variability in growth rates and ammonium assimilation could be measured in chemostat-grown bacterial cells. However, these techniques generally have low sample throughput, demanding sample preparation requirements, and can be costly in terms of time and/or money invested per cell, all of which can limit the scale of population surveys.

Raman microspectroscopy is amenable to single-cell applications and is complementary to MAR-FISH and nano-SIMS-FISH. Raman microspectroscopy has the advantages of non-destructively yielding intracellular molecular information, of requiring minimal sample preparation, and enabling rapid interrogation of many preserved or live cells. Recent advances in Raman microspectroscopic technology have dramatically broadened its microbiological applications (Brehm-Stecher and Johnson, 2004; Wagner, 2009; Huang et al., 2010; Wang et al., 2016). For example, Raman spectra of single cells have revealed metabolic histories and species identity, through characterization of an organism's macromolecular composition (e.g., Huang et al., 2004, 2007a; Hermelink et al., 2009; Hall et al., 2011). Huang et al. (2007b) demonstrated that intensity ratios of specific wavenumbers within Raman spectra from individual bacteria varied quantitatively with amount of ^13^C-glucose available. Furthermore, those cells were phylogenetically identifiable by FISH probing (SIP-Raman-FISH). Li et al. (2012) recently demonstrated that assimilation of ^13^C-enriched dissolved inorganic carbon (DIC) by individual photoautotrophic cells can be accurately quantified from wavenumber shifts in resonance Raman (SCRR) spectral peaks emanating from carotenoid pigments. Carotenoids are excellent target analytes because all photoautotrophic microbial taxa produce at least one form as accessory light-harvesting pigments or as protection against reactive oxygen species (Garcia-Asua et al., 1998). They are easily resolved by resonance Raman scattering, which increases photon scattering efficiency over spontaneous Raman scattering by at least a 1,000-fold by using laser excitation within the electronic transition frequency band of the analyte (e.g., Taylor et al., 1990; Robert, 2009).

We present a refinement of the SIP-SCRR-FISH approach (Li et al., 2012) that now enables quantitative Raman spectrometric measurement of growth rates in individual photoautotrophic cells. We use this tool to examine growth as the ultimate expression of inter- and intraspecific trait variability. Cells from replicate isogenic *Synechococcus* sp. cultures provided with varying concentrations of ^13^C-bicarbonate were interrogated by SCRR through time course experiments to determine their degree of labeling from which single-cell growth rates were calculated and compared to independent measurements of population growth. ^13^C-labeled populations of *Synechococcus* sp. and the diatom, *Thalassiosira pseudonana*, growing at known, but different rates were mixed to demonstrate that SCRR could distinguish among them. Optimal experimental design, underlying assumptions, analytical precision, and intra-population heterogeneity are evaluated. This technique has the advantages of relatively high sample throughput, low analytical costs, and potential application to natural phytoplankton communities in field experiments.

## Materials and methods

### Media preparation and cultivation conditions

Phytoplankton cultures were grown in f/2 media (Guillard and Ryther, 1962) in which total inorganic carbon (C_T_ = CO_2_ + H_2_CO_3_ + ${\text{HCO}}_{3}^{-}$ + ${\text{CO}}_{3}^{2-}$) was either replaced or augmented with varying proportions of ^13^C-enriched bicarbonate [${\text{HCO}}_{3}^{-}$; *see Supplementary Material (SM) 1*]. The ^12^C- and ^13^C-bicarbonate solutions (Cambridge Isotope Laboratories, Inc. Andover, MA; 99% ^13^C, 97% chemical purity) were prepared as 0.4 M working stocks. Nutrient and bicarbonate solutions were aseptically added to autoclaved filtered seawater (<0.22 μm = FSW) in 200-ml sealed septum bottles. C_T_ concentrations were then measured using a flow injection analysis system and compared to known C_T_ standards (Hall and Aller, 1992).

For the SIP experiment examining isotopic end-members, cultures of the cyanobacterium, *Synechococcus* sp. (RS9916), were grown at natural ^13^C abundances and at conditions under which 96% of the C_T_ was replaced with ^13^C-bicarbonate by pH manipulation (see *SM 1*). For the SIP calibration experiment, complete f/2 media was augmented with sterile bicarbonate solutions with varying ^13^C abundances (*f*_media_ = ^13^C_media_/(^12^C_media_ + ^13^C_media_)) and a mean final C_T_ of 3.78 ± 0.10 mM C (2 mM added). To minimize variations in growth conditions, C_T_ was kept constant while *f*_media_ was manipulated. Nominal (gravimetric) *f*_media_ determinations (0.011, 0.10, 0.20, 0.30, 0.40, and 0.50) were corrected to 0.011, 0.11, 0.22, 0.32, 0.43, and 0.54, respectively, based on actual *f*_media_ measured by isotope ratio mass spectrometry (IRMS) at the UC Davis Stable Isotope Facility. After inoculation, each septum bottle was attached to its own venting system permitting atmospheric exchange of all gases, except CO_2_. Modifications of Li et al.'s (2012) venting system were as follows: Ascarite II™ (Thomas Scientific®) replaced NaOH pellets as our CO_2_ trap in open 30 ml syringe barrels, capped with polyester fiber, and connected to 0.2 μm in-line filters that were joined to septum bottles by 1/8” (O.D.) stainless steel swan neck tubes. The swan neck replaced vertical tubes to prevent condensate from the CO_2_ trap leaking into septum bottles during extended incubations and contaminating samples. Cultures were subsampled through time while incubating at 20°C on a rotating platform that assured uniform light exposure (48–63 μmol quanta m^−2^ s^−1^) during a 12:12 h light/dark cycle.

Directly evaluating the SIP-SCRR method's ability to measure varying *f*_cell_ or growth rate in natural phytoplankton assemblages is difficult, if not impossible, without an independent method to measure non-uniform growth rates among the assemblage members. Therefore, a *Synechococcus* sp. assemblage was constructed by mixing equal volumes of 12-days cultures from six different *f*_media_ ratios. These cells were ostensibly growing at the same rate, but had different *f*_cell_ signatures due to exposure to different *f*_media_, essentially mimicking a mixed assemblage with varying growth rates. In a second experiment to compare contemporaneous slow-growing (*Synechococcus* sp.; mean g = 4.12 days) and fast-growing (*T. pseudonana*; mean g = 1.44 days) populations, cultures were grown in parallel at natural (0.011 *f*_media_) and elevated ^13^C abundances (0.48 *f*_media_) and subsampled through time. SCRR filters were prepared from mixtures of these cells as a mimic of a heterogeneous environmental sample.

### Population growth in SIP experiments

Daily change in *in vivo* chlorophyll *a* fluorescence was used to measure population growth in all treatments. After gentle agitation, triplicate 200-μl subsamples were assayed in a Turner Designs® Aquafluor™ fluorometer, calibrated according to manufacturer's instructions. When required, subsamples were diluted with sterile f/2 to remain below 80% detector saturation. Population growth rates (μ_pop_) were calculated from arbitrary fluorescence units (AFU) either as the regression slope of ln AFU vs. time for the entire exponential growth phase (mean μ_pop_) or as the difference between neighboring ln AFUs within the time course (instantaneous μ_pop,inst_ = [ln AFU_t+1_–ln AFU_t_]/[(t+1)–t]). Direct microscopic cell counts in the control sample confirmed that AFU values were highly correlated with cell concentrations (*r*^2^ = 0.97, *p* < 0.001) during exponential growth phase, signifying that ΔAFU/Δt is a reliable proxy for population growth under our experimental conditions.

### SCRR sample preparation from SIP experiments

For SCRR microspectrometry sampling, volumes removed (0.25—5.00 ml) were adjusted to obtain cell densities of 20–50 cells per microscope field and replaced with N_2_ gas to prevent a partial vacuum within the incubation bottles. After vortexing subsamples, cells were collected on 25 mm 0.2 μm polycarbonate membranes (Millipore® GTTP™) and rinsed with phosphate-buffered saline and filtered to dryness. Membranes were then air-dried in Millipore® PetriSlides™ and stored at −20°C.

In early experiments, potential interference of Cy3 fluorescent oligonucleotide probes with SCRR data acquisition was evaluated on membrane subsamples subjected to FISH. Membrane wedges were hybridized with a general bacterial probe (EUBMIX: Amann et al., 1990; Daims et al., 1999) in 35% formamide for 2 h and washed with buffer for 30 min and stored at −20°C until analysis (Pernthaler et al., 2001).

Although polycarbonate membranes are widely used for FISH and other fluorescence microscopies, these and most other types of filters and membranes produce contaminating Raman emissions (Raman-active). Likewise, borosilicate glass slides and cover slips and most mounting fluids (Citifluor™, Vectashield™, Cargille® Type A™ immersion oil, and glycerol) are Raman-active, water being the exception. Therefore, we developed a technique to transfer cells from polycarbonate membranes to mirror-finished 304 stainless steel slides (1 × 3 × 0.0235″) supplied by Stainless Supply® (Monroe, NC USA). Prior to use, slides were sequentially washed in an ultrasonic bath using acetone, isopropanol, and methanol, followed by MilliQ™ water rinsing at each step, and then air-dried.

To prepare both probe-hybridized (FISH) and non-hybridized cells for SCRR, samples were transferred to cleaned metal slides using a modified filter-transfer-freeze (FTF) technique (Hewes and Holm-Hansen, 1983). Briefly, each membrane wedge was placed sample-side down on a small droplet of sterile, particle-free MilliQ™ water (2–5 μl depending on wedge size) and the slide was placed on an aluminum cooling (−80°C) block. The membrane was peeled from the steel surface immediately after freezing, leaving most cells frozen to the slide, which was then air-dried in the dark (Suter, 2017). Transfer efficiency of *Synechococcus* cells was determined microscopically on filter wedge replicates; one set analyzed prior to freeze transfer and the other afterwards.

### Single-cell resonance Raman microspectrometry

SCRR measurements were performed using a Renishaw® inVia™ confocal Raman microspectrometer configured with a modified upright Leica® DM2700™ fluorescence microscope, a computer-controlled motorized XYZ stage (0.1 μm step size), 514 nm Ar^+^ ion laser, and 1,040 × 256 CCD Peltier-cooled detector. After cells were manually targeted by mouse clicks on a Cy3-fluorescent or autofluorescent digital image, the automated stage centered each cell under the laser beam for Raman data acquisition. For each sample, 25–40 cells (0.5–1.8 μm equivalent spherical diameters) were individually interrogated at 10% laser power (92 ± 7 μW at sample) through a Leica® dry 100 × (NA = 0.90) objective lens which produced a spot diameter of 0.7 μm. Spectra were obtained using a 65 μm slit centered at 1,752 μm, a 1,800 line/mm diffraction grating, aligned to wavenumber region between ~400 and 2,000 cm^−1^ (1,350 cm^−1^ center) and acquisition of two 1 s CCD detector exposures.

### Spectral analysis

Spectra were processed using Renishaw's® Wire 4.1™ software by first subtracting baselines using the software's standard best fit polynomial algorithm, and its intensity normalization function to standardize all spectra to a maximum peak value of 1, because cell-to-cell Raman scattering intensities are variable. Effects of ^13^C enrichment on behavior of dominant resonance Raman (RR) peaks were evaluated by two methods; peak-picking and curve-fitting. For peak-picking, center positions and widths of dominant spectral peaks were simply tabulated by the software and recognition thresholds were adjusted manually when required.

For curve-fitting analyses, two of the dominant RR peaks, usually positioned at ~1,521 and 1,157 cm^−1^, were deconvolved using Wire 4.1's function with our own empirical scripts. Peak shape and position are controlled by relative contributions of each isotopologue bond (e.g., ^12^C^12^C, ^12^C^13^C, ^13^C^13^C) to total peak area (*see SM 2*). Center positions ($\langle \Delta \tilde{\nu }\rangle$ = wavenumber, cm^−1^) for pure ^12^C^12^C and ^13^C^13^C isotopologues were estimated from the regression intercept and slopes of calibration curves [e.g., ν (C = C) vs. *f*_*cell*_], respectively. Center positions for the intermediate isotopologue peaks were estimated as the midpoint between the end-members. After local baseline correction, contribution of each isotopologue to triplet peak area was determined by allowing peak positions to float ± 1 cm^−1^ and peak widths to float ± 10 cm^−1^ and by running a full Voigt fit routine (floating Lorentzian and Gaussian contributions to peak form) for 5,000 iterations or a <0.00001 tolerance (difference between reduced chi-square values of two successive iterations), whichever came first. The curve fit function continuously compared the sum of the isotopologues' areas (synthetic peak) to observed data and reported the reduced Chi square values (goodness of fit).

### Determination of cell labeling and single cell growth rates

Since CO_2_ in the septum bottles is not allowed to exchange with the atmosphere, the isotopic signature of autotrophic cells (*f*_cell_) must predictably approach that of the media's DIC (*f*_media_, scaled for isotopic fractionation, α) with every cell division. Fractional labeling of populations was thus computed as follows.

To estimate α, an average δ^13^C value of −23%0 for marine plankton and a mean δ^13^C value of +1.5%0 for the ocean's mixed layer C_T_ pool were selected from global summaries (Goericke and Fry, 1994; Hoefs, 2009). Cellular ^13^C fractionation, α_cell−DIC_, was calculated from the isotope ratios (R = ^13^C/^12^C) of phytoplankton and seawater DIC using Equation (1).

(1) $$\begin{array}{l} \alpha_{cell-DIC}=\frac{R_{cell}}{R_{DIC}}=\frac{\delta^{13}C_{cell}+1000}{\delta^{13}C_{DIC}+1000}=\frac{-23+1000}{+1.5+1000}=0.976 \end{array}$$

Values for *f*_*cell*_ in specific samples were calculated via Equation (2), with uncertainties propagated from individual terms (*see SM 3 and 4 for derivations of equations*).

(2) $$\begin{array}{l} f_{cell}=\frac{\alpha f_{media}}{1+\left(\alpha -1\right)f_{media}}\\ +\left(\frac{\alpha f_{o}}{1+\left(\alpha -1\right)f_{o}}-\frac{\alpha f_{media}}{1+\left(\alpha -1\right)f_{media\;}}\right)e^{-nln\left(2\right)} \end{array}$$

where *f*_o_ = ancestral fractional isotopic signature of the media (0.011 ^13^C) and *n* = number of generations completed in the presence of enriched DI^13^C, which was calculated by Equation (3) using the population's specific growth (μ_*pop*_, day^−1^) measured by *in vivo* fluorescence.

(3) $$\begin{array}{l} n=\frac{t}{g}=t\frac{\mu_{pop}}{\ln\left(2\right)} \end{array}$$

where *t* = time elapsed (days) and *g* = generation time (days).

Single-cell growth (μ_sc_) can be calculated independently of population growth data if the *f*_cell_ of individual cells can be measured. SCRR enables such determinations if a spectral feature, such as mean peak wavenumber ($\langle \Delta \tilde{\nu }\rangle$) for the ν(C = C) or ν(C-C) bonds in carotenoids varies predictably with *f*_cell_. This relationship can be utilized according to Equation (4).

(4) $$\begin{array}{l} \langle \Delta \tilde{\nu }\rangle \approx b_{0}+b_{1}f_{cell} \end{array}$$

After determining the values of b_0_ and b_1_ experimentally, the number of generations (n) completed after spiking samples with DI^13^C can be calculated from measurements of $\langle \Delta \tilde{\nu }\rangle$ (Raman), *f*_media_, and *f*_o_ with Equation (5). Once *n* is derived, *g* or μ_sc_ is calculated from Equation (3).

(5) $$\begin{array}{l} n\approx \frac{1}{\ln\left(2\right)}\ln\left(\frac{f_{media}-f_{o}}{f_{media}-\left(1+\left(\alpha -1\right)f_{media}\right)\frac{{\langle \Delta \tilde{\nu }\rangle -b}_{o}}{\alpha b_{1}}}\right) \end{array}$$

### Statistics

Descriptive statistics, linear regressions, and analyses of variance (Kruskal-Wallis, Tukey, Dunn's and Holm-Sidak pair-wise comparison methods) were performed using SigmaPlot™ 13.0 software (Systat Software Inc.®). Graphics were produced by either Renishaw® Wire 4.1™ or SigmaPlot™ 13.0 software.

## Results

### SIP experimental design

For SIP experimental design, it was initially critical to establish whether DIC replacement or augmentation is preferable, to determine how much ^13^C-bicarbonate tracer is required, and then to accurately establish the value of *f*_media_. In principle, a known amount of the C_T_ pool must be removed before replacing with sufficient ^13^C-bicarbonate to return media to the original C_T_ pool size. Among published DIC replacement strategies, such as microwaving (Li et al., 2012), N_2_ purging, and pH manipulation, only pH manipulation adequately constrained the C_T_ and *f*_media_ terms (*see SM 1*).

DIC replacement may be suitable for experiments with cultivated populations in synthetic media, but is clearly inappropriate for experiments with natural field assemblages. DIC augmentation requires less manipulation and therefore potentially introduces fewer artifacts. More than doubling C_T_ depressed the pH of filtered seawater (FSW) by only 0.23 units (Table S1). In this study, C_T_ concentrations established gravimetrically from DIC additions closely agreed with those determined by flow injection analysis and by mass spectrometry, so the C_T_ and *f*_media_ terms were very well-constrained in experiments presented below (Table S2), which is absolutely essential for accurate growth calculations. Accordingly, experiments reported here used media augmented with the same C_T_ but varying *f*_media_, and pH was titrated back to 8.0 prior to inoculation with 0.1N NaOH (SM1), unless otherwise noted.

### SIP-Raman-FISH spectra

**Figure 1:**
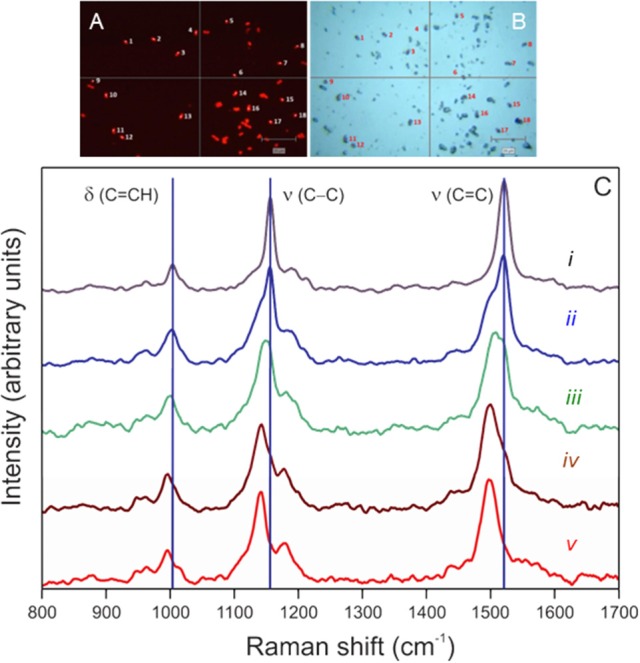
Examples of resonance Raman spectra obtained from EUBMIX-hybridized (Cy3) *Synechococcus* sp. (RS9916) cells (0.5–1.8 μm in diameter) pictured in fluorescence (Y3 ET, k—545 excitation/610 emission) **(A)** and bright-field **(B)** images. Cells manually targeted in the microscope field by the computer mouse are numbered, then automatically revisited under laser illumination for Raman interrogation. **(C)** Single-cell spectrum *i* was obtained from a culture grown on natural ^13^C abundances (*f*_media_ = 0.011 DI^13^C). Single-cell spectra *ii–v* were obtained from a culture in f/2 media augmented with 2 mM bicarbonate yielding a final fractional ^13^C-content of 54% (0.54 *f*_media_) after 3 (*ii*), 6 (*iii*), 12 (*iv*), and 24 (*v*) days of incubation. Vertical lines denote major peak positions in the control culture. Spectra were baseline-corrected, intensities normalized from 0 to 1, and smoothed using standard Renishaw™ Wire 4.1® routines.

While the identities of our cultures are known, phylogenetic identification of target cells by FISH enables application of SIP-SCRR to field samples. Cells hybridized against the Cy3-EUBMIX probe fluoresce brightly (Figure 1A) and can also be observed under bright-field illumination (Figure 1B). Cy3 fluorescence did not interfere with SCRR spectral acquisition when exciting within the carotenoid spectral absorption band (370–530 nm) at 514 nm (Figure 1C). Spectral responses to ^13^C enrichment of cellular biomass are evident in spectra *i–v* (Figure 1C). Spectrum *i* obtained in 2 s from a single cell grown in control media (*f*_media_ = 0.011) was dominated by three major peaks attributed to carotenoids. The 1,521 cm^−1^ peak has previously been assigned to ν (C = C) in-phase stretching, the 1,156 cm^−1^ to ν (C-C) in-phase stretching, and 1,004 cm^−1^ to a combination of δ (C = CH_3_) methyl deformation and δ (C-H) out-of-plane bending modes in β-carotene (Marshall and Marshall, 2010). Evolution of red-shifted peaks is evident as cells are grown in 54% DI^13^C for 3, 6, 12, and 24 days (spectra *ii–v*). Thus, cells actively growing photoautotrophically in DI^13^C are easily recognized by red-shifted positions of any of these three SCRR peaks.

### Quantitative application of single-cell resonance Raman microspectrometry

To evaluate whether SCRR microspectrometry can accurately quantify fractional labeling of individual photoautotrophic cells (*f*_cell_), parallel time course incubations of *Synechococcus* sp. were conducted under varying *f*_media_. Populations maintained exponential growth for the first 18 days at a mean rate of μ_pop_ = 0.220 ± 0.002 day^−1^ (g = 3.20 ± 0.03 days) for all treatments as measured by *in vivo* fluorescence (Figure S1) and thus were unaffected by *f*_media_ values between 0.011 and 0.54 (*p* > 0.98; Kruskal-Wallis one-way ANOVA). Thus, the range of *f*_media_ used did not appear to introduce artifacts to the SIP-SCRR experiments and theory dictates that all populations would incorporate ^13^C into intracellular pools in proportion to their particular *f*_media_.

**Figure 2:**
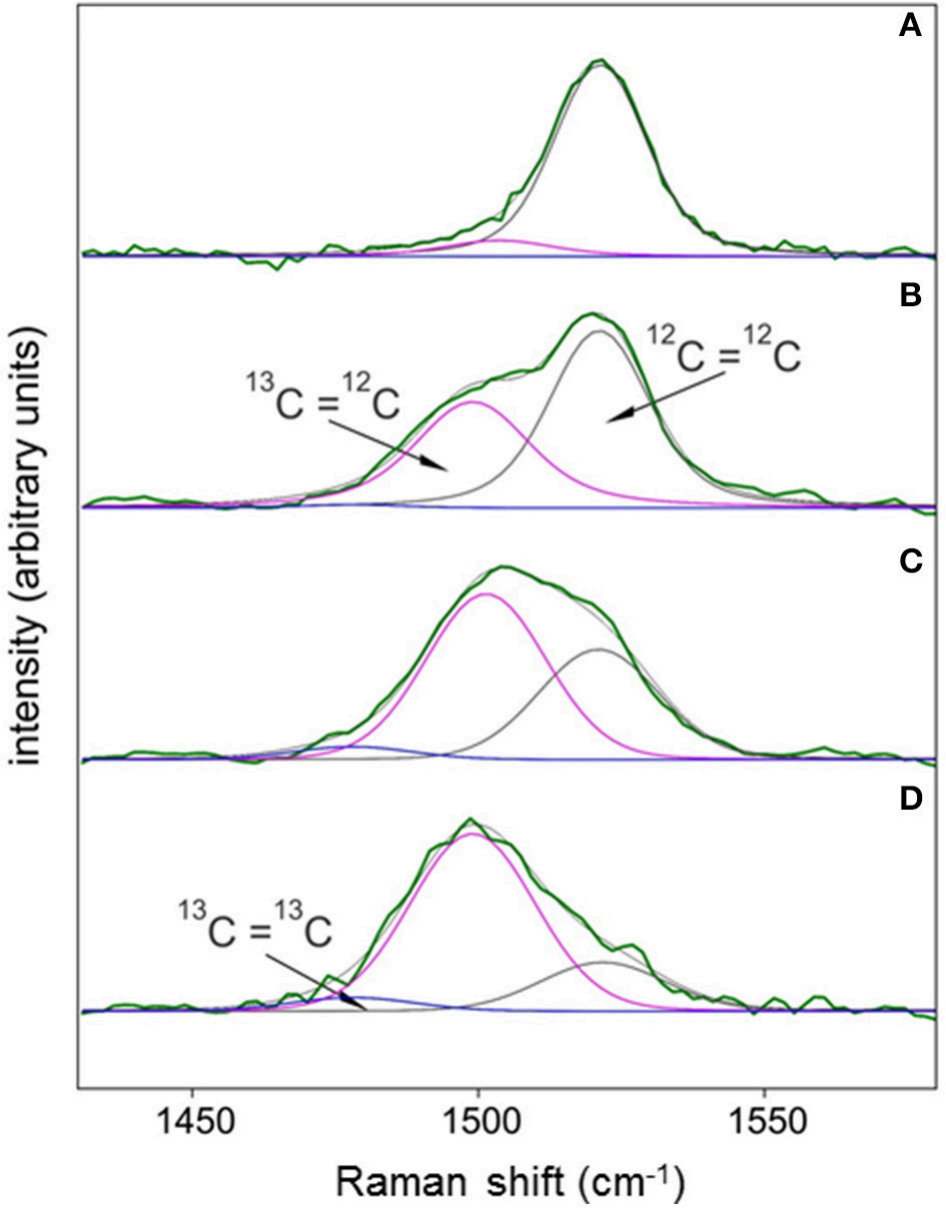
Examples of the varying contributions of ^12^C=^12^C, ^12^C=^13^C, and ^13^C=^13^C isotopologues to the shape, position, and areas of the ν (C = C) Raman spectral peak for carotenoids of cells assimilating varying amounts of DI^13^C. Each Raman spectrum was obtained from individual cells grown in either 0.011 *f*_media_ **(A)** or 0.54 *f*_media_ for 3 **(B)**, 6.2 **(C)**, or 9.2 **(D)** days. Spectra were subjected to local baseline subtraction (1,360–1,660 cm^−1^), intensity normalization (0–1), and a full Voigt curve-fitting routine (convolution of Lorentzian and Gaussian profiles) with 5,000 iterations or a 0.00001 tolerance using Renishaw™ Wire 4.1® software. Center positions for each of the three isotopologues were constrained within narrow consensus ranges (± 1 cm^−1^) determined from regression coefficients presented in Figure 5. Isotopologue peak widths and symmetries were allowed to float to optimize curve fits.

**Figure 3:**
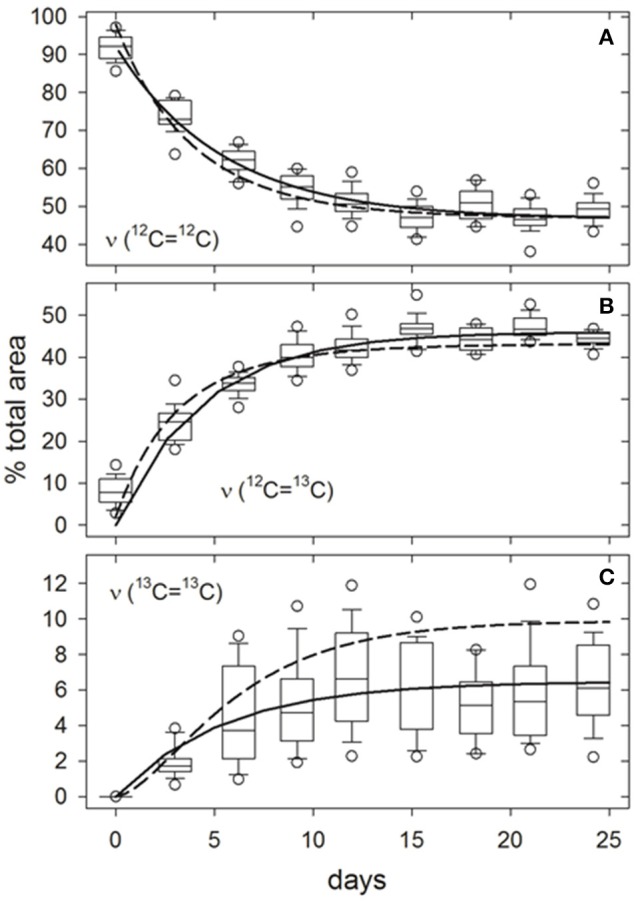
Example of growth-dependent variations in the relative proportions of ν (^12^C=^12^C) **(A)**, ν (^12^C=^13^C) **(B)**, and ν (^13^C=^13^C) **(C)** isotopologue bond peak areas to the total ν (C = C) Raman peak area at ~1,521 cm^−1^ for carotenoids in *Synechococcus* sp. (RS9916) cells grown in 0.32 *f*_media_. Boxes represent the interquartile range (25–75th percentiles) for peak areas in spectra from 25 ± 3 individual cells. Internal horizontal lines, whiskers, and circles are medians, 10 to 90th, and 5 to 95th percentiles for all observations, respectively. Solid curves are hyperbolic fits to all observations (*N* = 250 cells). Broken lines are responses predicted from arguments presented in SM 2 and SM 3, particularly Equations (S11, S20).

SCRR spectra were subjected to Renishaw's® Wire 4.1™ curve-fitting routine to illustrate how resonances from ^12^C^12^C, ^12^C^13^C, and ^13^C^13^C bonds (isotopologues) contribute to the total area, position, and shape of peaks as cells become ^13^C-enriched. As an example, cells grown in *f*_media_ = 0.011 (Figure 2A) and 0.54 for 3–9 days illustrate the effects of ^13^C enrichment on the 1,521 cm^−1^ (ν (C = C)) peak (Figures 2B–D). During early stages of labeling (Figures 2B,C), the triplet peak widens with a distinct shoulder that disappears as ^12^C=^13^C replaces ^12^C=^12^C (Figure 2D). This transition causes red-shifting of the triplet peak's mean resonance ($\langle \Delta \tilde{\nu }\rangle$) and eventually leads to bandwidth narrowing (Figures 1, 2D). Curve-fitting skill is illustrated by the coherence between fit curve solutions (gray lines; Figure 2) and observed data (green lines; Figure 3). The mean reduced Chi Square was 1.00 ± 0.25 (S.D.) for the entire 0.54 *f*_media_ time course (*N* = 172 cells).

Curve-fitting analysis of spectra from the entire *f*_media_ = 0.32 time course illustrate that the proportion of underlying peak areas attributed to ^12^C=^12^C bonds decreased asymptotically to ~50%, while those of ^12^C=^13^C and ^13^C=^13^C bonds increased asymptotically to ~44 and ~6%, respectively, as ^13^C was incorporated into cellular biomass (non-linear least-squares regression, solid lines, Figure 3). Observed changes are consistent with our model for ^13^C-incorporation at random positions in a representative carotenoid molecule (β-carotene) (dashed lines, Figure 3; see SM 2 and SM 3 for derivation). In the present example, fractionation would theoretically limit *f*_cell_ to a maximum value of 0.31, at which the expected proportions of ^12^C=^12^C, ^12^C=^13^C, and ^13^C=^13^C bonds and their associated peak areas would approach 48, 43, and 10%, respectively (Equations S11, S20). However, unlike ^12^C=^12^C and ^12^C=^13^C bonds, the observed and predicted proportions of ^13^C=^13^C bonds were significantly different after 24 days (Figure 3C). We attribute this discrepancy to lower curve-fitting skill caused by the baseline correction method employed, which could undoubtedly be improved upon. The same time/growth-dependent trends in isotopologue contributions to the (ν (C = C)) peak area were observed at all levels of DI^13^C augmentation (not presented). Curve-fitting skills for the isotopologues contributing to the 1,156 cm^−1^ (ν (C-C)) and 1,004 cm^−1^ (δ (C = CH)) peak areas were less satisfactory due to baseline uncertainties and overlapping spectral features from other molecular bonds, particularly near 1,004 cm^−1^ (not presented).

**Figure 4:**
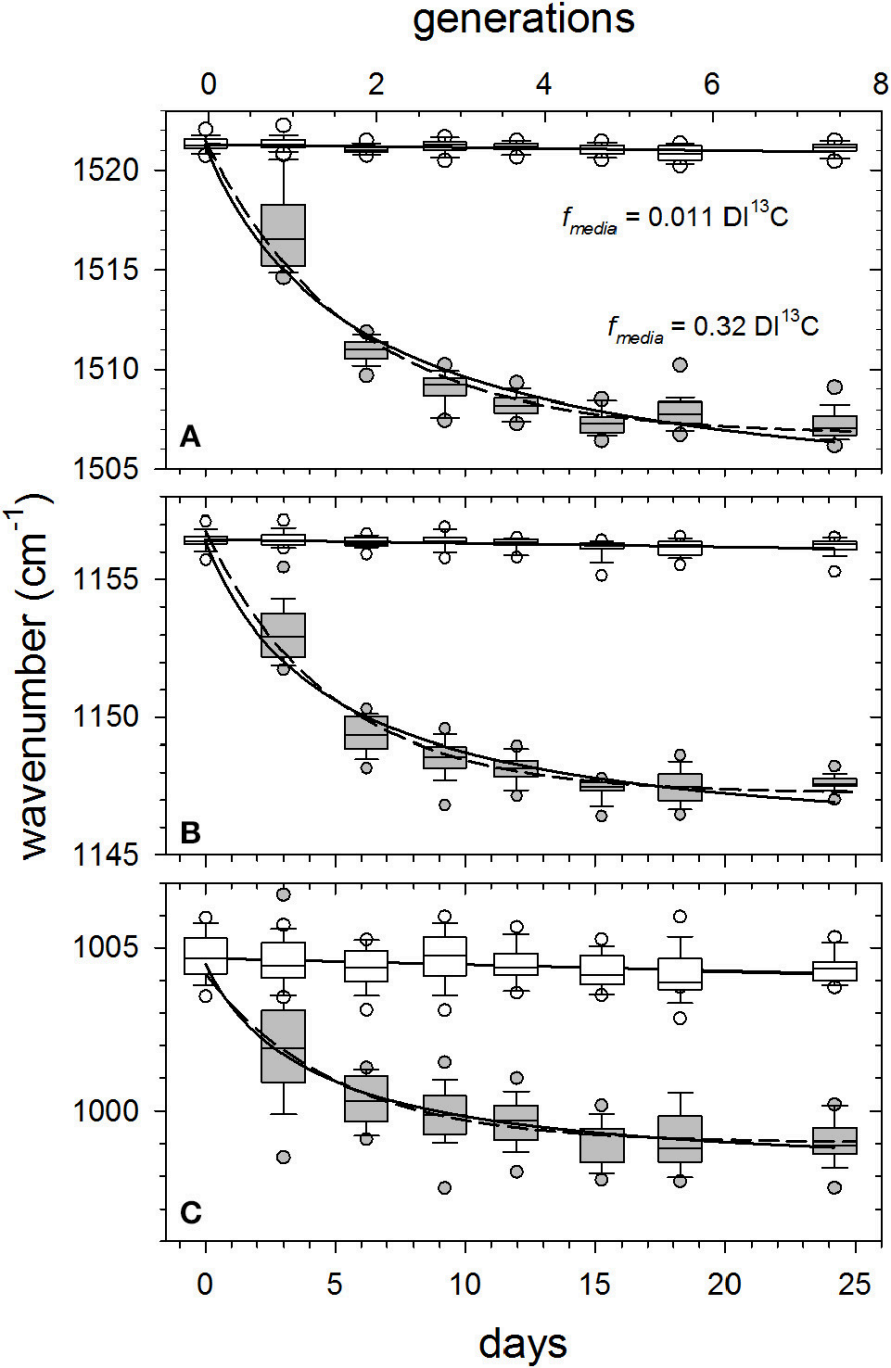
Examples of growth-dependent shifts in mean wavenumbers $\langle \Delta \tilde{\nu }\rangle$ of the major carotenoid resonance Raman spectral peaks as *Synechococcus* sp. (RS9916) cells grown in 0.32 *f*_media_ compared with 0.011 *f*_media_ controls. Generations (n) were calculated from generation times (g) presented in Figure S1 and incubation times (n = t/g). Box and whisker plot details are as described in Figure 3. Solid curves are hyperbolic fits to all observations (*N* = 325 cells). Dashed lines are theoretical predictions for a population isotopically equilibrating with its media based on wavenumber response to *f*_cell_ and generation time (*see SM 2 and SM 3 for derivation*). Solid horizontal lines are least square means of all observations in control samples. Peak assignments are **(A)** ν (C = C), **(B)** ν (C-C), and **(C)** δ (C = CH).

As an alternative to curve-fitting peak areas as a function of time (e.g., Figure 3), we simply compared software-determined center positions ($\langle \Delta \tilde{\nu }\rangle$) of major peaks in randomly selected cells (*N* = 25 cells sample^−1^) for all DI^13^C-augmented cultures to the control (0.011 *f*_media_). Figure 4 illustrates typical asymptotic responses of the three major peak wavenumbers as functions of growth in the *f*_media_ = 0.32 treatment. Declines in peak wavenumber (red shifts) are steep over the first 2 generations, slowing as they approach an asymptote in about 5–7 generations. Clearly, growth-dependent changes in mean Raman peak wavenumbers ($\langle \Delta \tilde{\nu }\rangle$) of the three carotenoid signature peaks observed in single-cells (boxes and whiskers) agree well with theoretical predictions (dashed lines) of red-shifting resonances for a population isotopically equilibrating with its media (*see SM 2 for derivation of prediction*).

**Figure 5:**
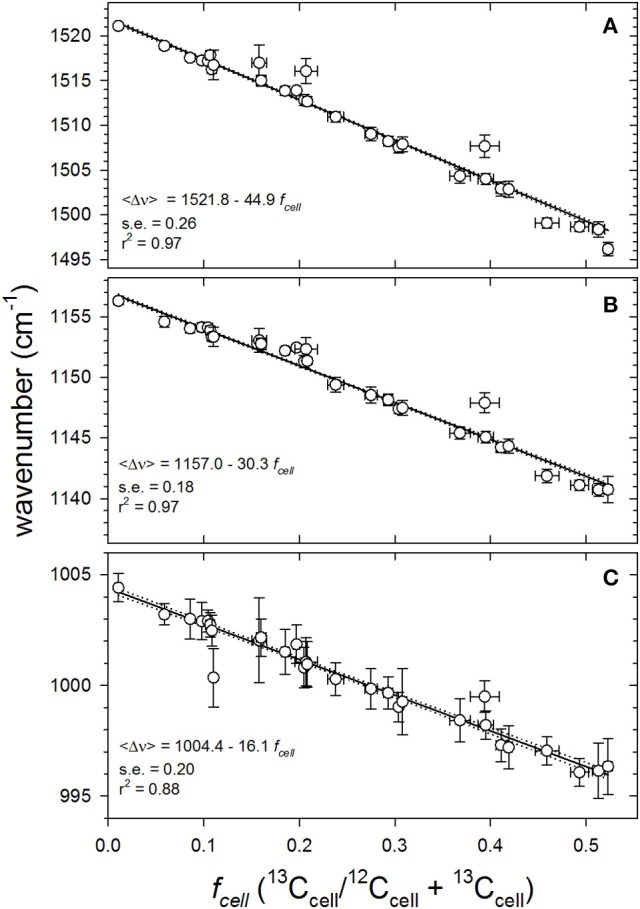
Quantitative responses of mean SCRR wavenumbers $\langle \Delta \tilde{\nu }\rangle$ of the three dominant carotenoid peaks to varying cellular fractional labeling (*f*_cell_) for all *f*_media_ (0.011–0.54) throughout exponential growth phase (0–18 days). Symbols and vertical error bars represent mean SCRR wavenumber observations ± 1 S.D., respectively. Horizontal error bars represent uncertainties propagated in calculating *f*_cell_ (*see SM 3*). Solid lines are empirical least squares linear regressions through individual data points (*N* = 836 cells). Dotted lines define the 99% confidence intervals of each slope (*b*_1_); **(A)** ν (C = C), **(B)** ν (C-C), and **(C)** δ (C = CH).

The most useful, generalized trend is obtained by constructing calibration curves directly comparing $\langle \Delta \tilde{\nu }\rangle$ with *f*_cell_. Knowing *f*_media_ and population growth (μ_*pop*_ or *g*) from independent chemical and fluorescence measurements (Figure S1) and assuming an isotopic fractionation factor (α) of 0.976 Equation (1), *f*_cell_ can be calculated from Equation (2) and Equation (3). Mean SCRR wavenumbers ($\langle \Delta \tilde{\nu }\rangle$) of the three major carotenoid peaks declined linearly across all values of *f*_cell_ examined; from natural ^13^C abundances (*f*_cell_ = 0.0107) to an *f*_cell_ of ~0.53 (Figure 5). The regressions' coefficients of determination (*r*^2^ = 0.88–0.97), the slopes' standard errors (s.e. = 0.18–0.26) and 99% confidence intervals demonstrate well-constrained relationships between *f*_cell_ and ($\langle \Delta \tilde{\nu }\rangle$). Collectively, the robust linear regressions and their agreement with theory suggest that *f*_cell_ can be reliably approximated by simply measuring $\langle \Delta \tilde{\nu }\rangle$.

Analogous examination of the curve-fitting approach across the entire *f*_cell_ range yielded results consistent with peak position analysis, i.e., contributions of the individual isotopologues to triplet peak area varied predictably with cell ^13^C-content (not presented). However, scatter evident in isotopologue peak contributions, largely due to variable curve-fitting skill, renders isotopologue peak area analysis less suitable than the highly predictable relationship between $\langle \Delta \tilde{\nu }\rangle$ and *f*_cell_ for quantitative applications. Nevertheless, empirical curve-fitting results conform to theory and illustrate the mechanics behind red-shifting peak wavenumbers.

### Variability and sensitivity of SCRR

Scatter in SCRR microspectrometric data emanate from both natural cell-to-cell variability in ^13^C-content inherent to different growth phenotypes and from analytical uncertainty. Our sample size (25–40 cells) was adequate to distinguish between time points and treatments and to represent intra-population variability (e.g., Figures 3–5). If required, uncertainty could be reduced somewhat with larger sample sizes. Potential sources of analytical error in Raman microspectrometry include: improper spectrometer calibration, beam misalignment, varying laser intensity, poor beam focusing, variable target geometry, and contaminating materials associated with target cells. However, current instrumentation and best practices eliminate or minimize most of these issues. Concerns about small variations in focus and target geometry are irrelevant because they primarily affect emission intensity which is not critical to our analyses.

*Synechococcus* sp. cells perpetually grown on natural ^13^C/^12^C abundances serve as the best candidate for an authentic standard to assess analytical error. Table 1 summarizes spectral data obtained from all control cultures. Assuming ^13^C and ^12^C atoms are distributed at random positions in the carotenoid molecules, about 98% of all C = C bonds are predicted to be ^12^C=^12^C in populations grown in natural ^13^C abundances (*see SM 2*). In the dataset most amenable to curve-fitting [the ν (C = C) triplet], however, the ^12^C=^12^C isotopologue in all control cultures accounted for an average of 89 ± 9% of the ν (C = C) peak area with ^12^C=^13^C and ^13^C=^13^C isotopologues contributing the remaining 9 and 1%, respectively (Figure 2A, Table 1). Although within uncertainty of the predicted percentages, this large discrepancy likely results from our curve-fitting model's simplicity, uncertainties in absolute peak positions of the three isotopologues, baseline position, and peak broadening in condensed matter, all of which could contribute to curve-fitting inaccuracies.

**Table 1:** Analytical precision of single-cell resonance Raman spectral features assessed from 150 *Synechococcus* sp. (RS9916) continuously cells grown at natural ^13^C abundances (*f*_media_ = 0.011).

		**% of total area**		**δ (C = CH) 〈Δν〉**	**ν (C-C) 〈Δν〉**	**ν (C = C) 〈Δν〉**
	**^12^C=^12^C**	**^12^C=^13^C**	**^13^C=^13^C**	**Center (cm^−1^)**	**Center (cm^−1^)**	**Center (cm^−1^)**
Minimum	52.9	0.0	0.0	1002.6	1155.1	1520.2
Maximum	100	47.1	4.6	1006.1	1157.2	1522.4
Mean	89.0	10.1	1.0	1004.4	1156.3	1521.1
*SD*	8.9	9.4	1.0	0.65	0.29	0.34
%CV^a^	9.9	93.0	97.9	0.06	0.03	0.02
99% C.I.^b^	±1.9	±2.0	±0.2	±0.14	±0.06	±0.07

a *%CV = coefficient of variation = standard deviation × 100/mean*.

b *99% confidence intervals*.

In contrast, the mean wavenumbers ($\langle \Delta \tilde{\nu }\rangle$) of all three carotenoid signature peaks were practically invariant in natural ^13^C abundance control populations, and indicate the technique's analytical reproducibility (Table 1). Precision for $\langle \Delta \tilde{\nu }\rangle$ peak position determinations was excellent; the 99% confidence intervals around the 1004.4, 1156.3, and 1521.1 cm^−1^ means were ±0.14, 0.06, and 0.07 cm^−1^, respectively for 150 *Synechococcus* cells. From these analyses, we conclude that $\langle \Delta \tilde{\nu }\rangle$ is a far more precise indicator of *f*_cell_ than isotopologue peak areas, and that the positions of the ν (C-C) and ν (C = C) peaks in control cultures are more reproducible than that of the δ (C = CH) peak.

Carotenoids in photoautotrophs have evolved a wide variety of chemical structures along diverging phylogenetic lines. Therefore, SCRR spectra from different species are likely to differ subtly. In the interest of developing a broadly applicable SCRR tool to ultimately determine growth rates in complex phytoplankton communities, available Raman spectral data for carotenoids were evaluated (Table 2). Among a variety of photoautotrophic microorganisms, the ν (C = C) and δ (C = CH) peak positions in cells growing at natural ^13^C abundances spanned 10.2 and 8.1 cm^−1^, respectively, while the ν (C-C) peak spanned just 2.4 cm^−1^ (Table 2). Furthermore, ν (C-C) peak wavenumbers observed in photoautotrophic protists were statistically indistinguishable from those of cyanobacteria (ANOVA *p*-value = 0.905), whereas the ν (C = C) and δ (C = CH) peak positions were more phylogenetically distinctive (Table 2). Therefore, even though the ν (C = C) peak had the broadest dynamic range and highest precision within our *Synechococcus* cultures, we chose the more phylogenetically conserved ν (C-C) peak for further quantitative analyses.

**Table 2:** Variability in major wavenumber positions (cm^−1^) for carotenoid peaks in photosynthetic microorganisms grown under natural stable isotope abundances.

**Source**	**ν (C = C)**	**ν (C-C)**	**δ (C = CH)/δ (C-H)**	**References**
β-carotene	1,518	1,157	1,010	Marshall and Marshall (2010)
**PROKARYOTES**				
*Synechocystis* sp. (PCC 6803)	1,517	1,156	1,004	Li et al. (2012)
*Synechocystis elongatus* (PCC 7942)	1,522	1,158	1,006	Li et al. (2012)
*Synechococcus* sp. (RS9916)	1,521	1,156	1,006	This study
Prokaryote Mean	1519.9	1156.5	1005.3	
*SD*	2.8	1.1	0.9	
%CV	0.18	0.09	0.09	
**EUKARYOTES**				
Arctic “AMA” microalgae	1,524	1,157	1,003	Li et al. (2012)
*Thalassiosira weissflogii* (Bacillariophyta)	1,526	1,157	1,010	This study
*Thalassiosira pseudonana* (Bacillariophyta)	1,526	1,156	1,010	This study
*Heterocapsa triquetra* (Dinoflagellata)	1,525	1,156	1,011	This study
*Aureoumbra lagunensis* (Pelagophyte)	1,527	1,158	1,009	This study
Eukaryote Mean	1525.6	1156.6	1008.5	
*SD*	1.2	0.9	3.2	
%CV	0.08	0.08	0.32	
ANOVA *p*-value^a^	0.006	0.905	0.250	

a *Kruskal-Wallis one way analysis of variance comparing prokaryotic and eukaryotic values. p-values exceeding 0.05 indicate no statistical difference between data sets*.

Theoretical sensitivity or limit of detection (LOD) of the mean SCRR wavenumber approach was calculated using the regression statistics from Figure 5 and the formula, LOD = 3 *S*_P_*/b*_1_, where *S*_P_ is the pooled standard deviations (±0.305 cm^−1^) of $\langle \Delta \tilde{\nu }\rangle$ observed at each value of *f*_cell_ and *b*_1_ = 30.3 cm^−1^/*f*_cell_ (Winefordner and Long, 1983). This calculation yielded a LOD of 0.03 leading us to conclude that a Δ *f*_cell_ of ~3% is detectable by our technique.

### Single-cell growth rates determined by SCRR

**Figure 6:**
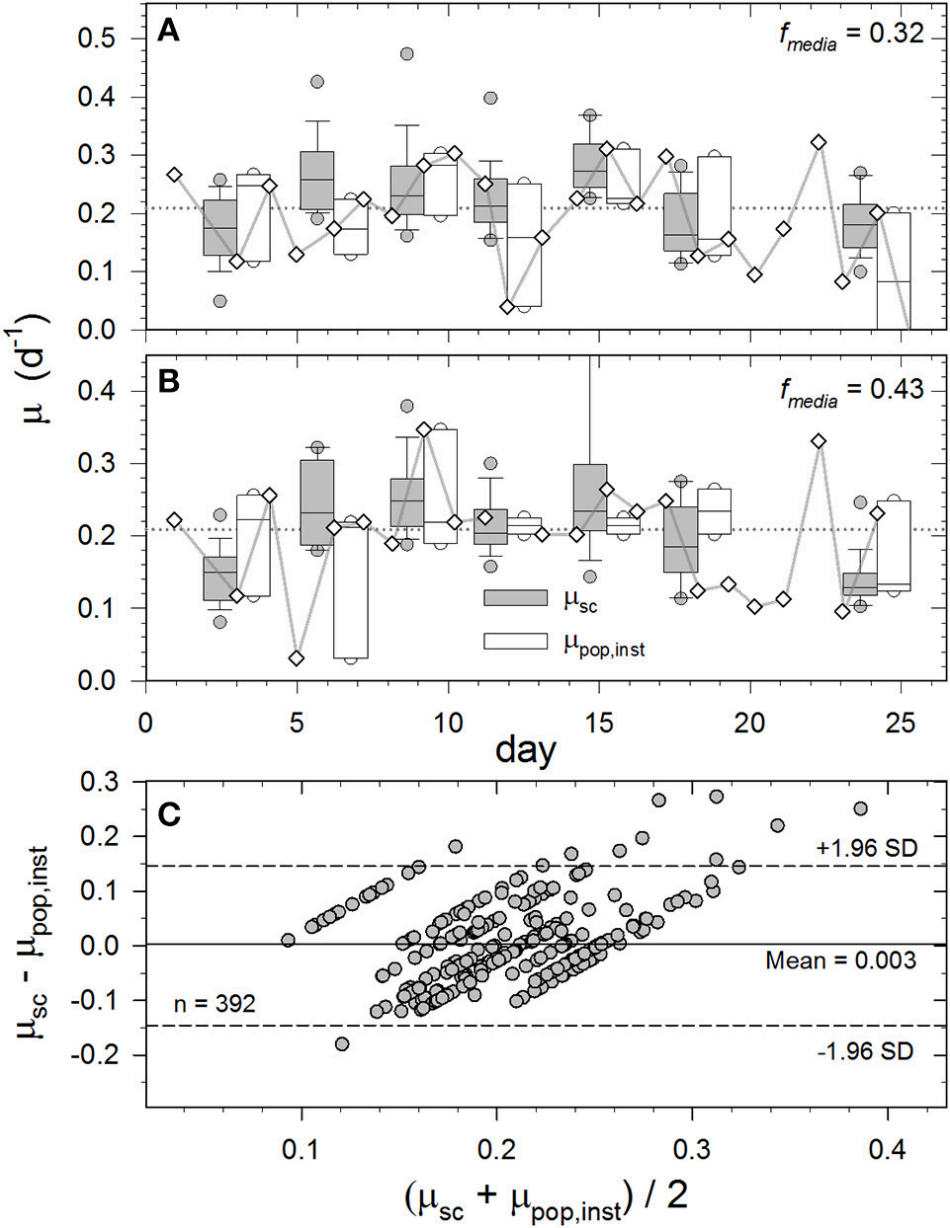
Temporal variations in single-cell growth rates (μ_sc_) calculated from SCRR peak positions $\langle \Delta {\tilde{\nu }}_{C-C}\rangle$ and daily population growth rates (μ_pop,inst_–diamonds) based on *in vivo* fluorescence measurements (Figure S1) for cultures incubated in *f*_media_ = 0.32 **(A)** and 0.43 **(B)**, and Bland and Altman (1986) plot comparing results from both methods and treatments **(C)**. See Materials and Methods for calculations. Box and whisker plot details are as described in Figure 3. Shaded boxes represent single-cell growth rates (μ_sc_) and open boxes represent daily population growth rates (μ_pop,inst_) at *t*_x−1_*, t*_x_, and *t*_x+1_. Horizontal broken line is mean μ_pop_ over entire exponential growth phase (0–18 days). **(C)** Differences between μ_sc_ and μ_pop,inst_ compared to their mean values to assess their agreement. Broken horizontal lines represent the 95% confidence intervals (±1.96 standard deviations).

Autotrophs will necessarily approach the isotopic signature of DI^13^C-augmented media (*f*_media_) as a function of time, growth rate, and isotopic fractionation [Equation (2) and Equation (3)]. Therefore, the empirical linear relationship between $\langle \Delta \tilde{\nu }\rangle$ and *f*_cell_ (Figure 5) can be used to estimate single-cell growth rates from time course (e.g., Figure 4) or brief end-point incubations. To illustrate, 25 single-cell growth rates (μ_sc_) throughout the 0.32 and 0.43 *f*_media_ incubations are compared to proximal daily population growth rates (μ_pop,inst_) determined at t_x−1_, t_x_, and t_x+1_ (open boxes and whiskers Figures 6A,B). While mean μ_pop_ from 0 to 18 days was ~0.21 day^−1^ for both populations (broken horizontal lines), these relatively slow-growing photoautotrophs (g ≈ 3.3 days) exhibited considerable day-to-day oscillations in μ_pop,inst_. (diamonds). We speculate that these oscillations result from intrinsic cell cycle periodicity and/or extrinsic variations in incubation conditions. Nonetheless, median growth rates computed over the entire exponential phase of *f*_media_ 0.32 and 0.43 cultures were 0.22 ± 0.07 and 0.20 ± 0.07 day^−1^ for single cells (Raman-derived) and 0.22 ± 0.07 and 0.22 ± 0.07 day^−1^ for populations (fluorescence-derived), respectively (Figures 6A,B). In fact, μ_sc_ computed from SCRR measurements between 0 and 18 d for the 0.11, 0.32, 0.43, and 0.54 *f*_media_ treatments were statistically indistinguishable from μ_pop,inst_ measured over the same period (*p* > 0.05; ANOVA). Inexplicably, μ_sc_ rates derived from the 0.22 *f*_media_ treatment were lower than those from μ_pop,inst_ measurements in all but the 3 day sample (*p* < 0.05 ANOVA; not presented).

Both *in vivo* fluorescence and SIP-SCRR based measurements of specific growth rate are subject to errors that may contribute differing amounts to the observed variability. For comparison, the mean %CV for $\langle \Delta \tilde{\nu }\rangle$ values obtained by SCRR (± 0.05%; *N* = 861) within individual samples was two orders of magnitude smaller than that obtained by daily triplicate *in vivo* fluorescence measurements (± 3.7%; N = 144; S.E. = ± 0.23). The mean relative error for *f*_cell_ was ±2.0% (S.E. ± 0.25), which is controlled by uncertainties in *b*_0_ and *b*_1_ in Figure 5 and measurements of *f*_media_. CVs for μ_sc_ tend to be higher at lower *f*_media_ and shorter incubations times *(see SM 4 for discussion of uncertainties)*. Of the observed variance for μ_sc_ through time ($\bar{CV}$ = 29% ±2.4 S.E.) illustrated in Figure 6, more than 93% appears to be due to cell-to-cell heterogeneity in physiological state within these isogenic populations. Likewise, most variance observed in μ_pop,inst_ through time ($\bar{CV}$ = 64%±24 S.E.) is likely due to actual variations in cell division, rather than analytical imprecision. We note that *in vivo* fluorescence measurements were made at approximately the same time every day (±1.5 h). Consequently, samples were undoubtedly withdrawn at different points within the 3.3 days cell cycle of these populations.

An approach routinely used in biomedical studies for evaluating agreement between two independent methods of measuring a single variable is to visualize the distributions of the differences between the two measurements plotted against their means (Bland and Altman, 1986). The Bland-Altman plot is more appropriate than regression analysis when those independent measurements have small dynamic ranges that are surpassed by the measurements' observed variability. Distributions of observed μ_sc_—μ_pop,inst_ in the *f*_media_ = 0.32 and 0.43 at each time point are compared to (μ_sc_ + μ_pop,inst_)/2 in Figure 6C, where the μ_pop,inst_ term for each μ_sc_ was the mean determined at *t*_*x*−1_*, t*_*x*_, and *t*_*x*+1_. If the two measurement methods consistently produced identical results, all points would fall on the horizontal zero line. In the current case, the average difference is +0.003, meaning that on average the μ_sc_ method returns a 0.3% higher value than the μ_pop,inst_ method among all observations. The +1.96 and –1.96 boundaries (broken horizontal lines) represent the 95% confidence intervals, and 16 of the 17 outliers are positive, again illustrating that the μ_sc_ method tends to return slightly higher values than the μ_pop,inst_ method. Absolute accuracy of our μ_sc_ method cannot be determined from this analysis largely due to cell-to-cell variability and uncertainties over which of the daily μ_pop,inst_ measurements are most representative of a particular μ_sc_ time point. Nonetheless, the data distribution in Figure 6C illustrates that the vast majority of SIP-SCRR-derived growth rates are within the 95% confidence intervals of independently-measured population growth rates. Furthermore, the preceding error analysis suggests that most observed variability is attributable to real differences in growth activity and not to analytical error.

### SIP-SCRR-derived growth rates in mixed assemblages

**Figure 7:**
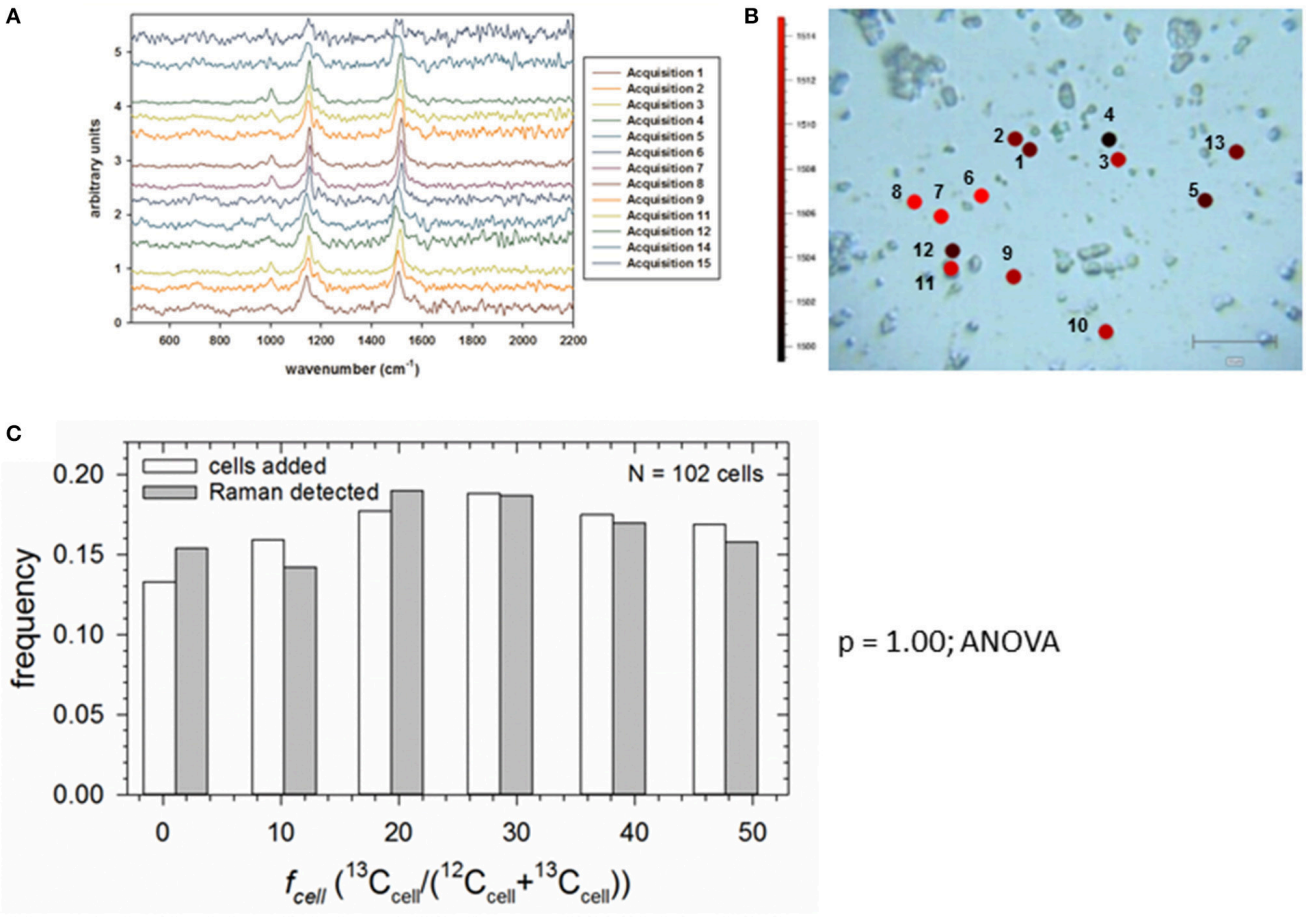
SCRR of an artificial assemblage constructed of known concentrations of *Synechococcus* sp. populations with distinct *f*_cell_ labeling. **(A)** Stacked SCRR spectra from cells 1–13. **(B)** Bright field image of cells 1–13 targeted for Raman interrogation in a single field superimposed with $\langle \Delta {\tilde{\nu }}_{C-C}\rangle$ wavenumber color codes. **(C)** Comparison of frequency of occurrence of cells added from each *f*_cell_ population (open bars) and those detected by SCRR (shaded bars).

Our final objective was to demonstrate that SIP-SCRR can measure growth rates of individual cells in a mixed photoautotrophic assemblage whose members are assimilating ^13^C-bicarbonate at contrasting rates. In the absence of an independent method to measure single-cell growth in natural communities, we constructed an artificial assemblage by dispensing equal volumes of *Synechococcus* cell suspensions from the six *f*_media_ treatments (Figure 5) at a single time point. Figure 7A presents the SCRR spectra obtained from 13 individual cells in the mixed assemblage (Figure 7B). After interrogating multiple fields, the statistical distribution of *Synechococcus* sp. cells with six distinctive ^13^C signatures added to the mixture were indistinguishable (ANOVA; *p* = 1.00) from those recognized by SIP-SCRR (Figure 7C). Therefore, morphologically identical cells with distinctive ^13^C signatures can be recognized and reliably quantified within an isotopically mixed assemblage by this method.

**Figure 8:**
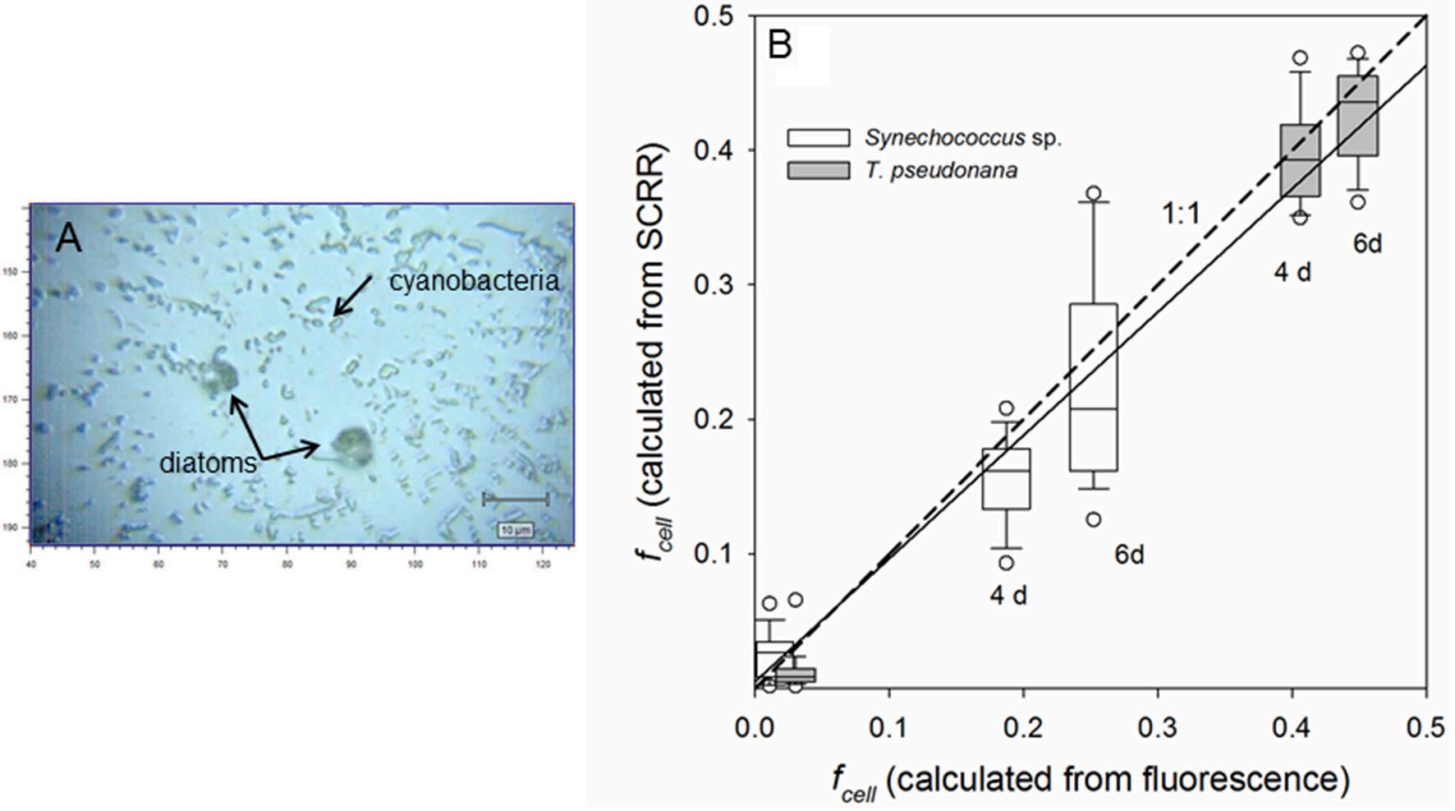
Bright-field image **(A)** and measurement of fractional labeling **(B)** of single cells in artificial assemblages through time. Parallel cultures of a fast-growing diatom, *Thalassiosira pseudonana* (μ_pop_ = 0.483 day^−1^) and a slow-growing cyanobacterium, *Synechococcus* sp. (μ_pop_ = 0.161 day^−1^) in labeled (*f*_media_ = 0.48) and unlabeled (*f*_media_ = 0.011) were subsampled through exponential growth phase, mixed, and prepared for SCRR interrogation. Values of *f*_cell_ computed from SCRR peak positions $\langle \Delta {\tilde{\nu }}_{C-C}\rangle$ and Equation (4) in individual cells are compared with *f*_cell_ computed from α, *f*_media_, population growth measured by *in vivo* fluorescence, and Equation (2) **(B)**. Solid line represents linear regression of all observations (*N* = 253 cells) including natural ^13^C abundance controls (*f*_cell_ ≈ 0.0107); *f*_cell_(SCRR) = 0.005 + 0.92±0.02 *f*_cell_ (fluorescence), *r*^2^ = 0.92.

To further evaluate application of this technique to phylogenetically diverse field assemblages, subsamples of parallel cultures of relatively slow-growing cyanobacteria and fast-growing diatoms were mixed at several time points. Grown in identical media (*f*_media_ = 0.011 and 0.48), the diatom, *T. pseudonana*, and cyanobacterium, *Synechococcus* sp., maintained exponential growth for 6 days, attaining population growth rates of 0.483 and 0.161 day^−1^, respectively. In Figure 8, the distribution of *f*_cell_ values computed by plugging SCRR observations of $\langle \Delta {\tilde{\nu }}_{C-C}\rangle$ into Equation (4) are compared to *f*_cell_ values predicted from α, *f*_media_, population growth measured by *in vivo* fluorescence of the cultures and Equation (2). The trend line computed from all cells (*N* = 253) spanning *f*_cell_ values of 0.0107 to 0.449 is not significantly different from the 1:1 line. These results demonstrate that within experimental error and intra-population variability, fractional ^13^C-labeling of single diatom and cyanobacterial cells computed from SIP-SCRR measurements closely agrees with fractional labeling of entire populations grown at natural and enhanced ^13^C-DIC abundances. Results presented in Figures 7, 8 support the proposition that single-cell growth rates within mixed natural photoautotrophic assemblages can be computed from SIP-SCRR measurements.

## Discussion

### Single-cell productivity techniques

Individual or agent-based ecological models suggest that intra-population variability in genetics, cell line history and microspatial geochemical heterogeneity can influence population responses to environmental forcing along multiple pathways and lead to divergent outcomes (e.g., Hellweger and Kianirad, 2007; Bolnick et al., 2011; Bucci et al., 2012; Fredrick et al., 2013). Empirical testing of such models is challenging because the requisite single-cell productivity measurements have been limited historically to a few laboratory techniques with cultures. For example, division of individual cells encapsulated in mineral oil has been followed through time microscopically (Dewan et al., 2012; Damodaran et al., 2015) and increasing mass of individual cells has been measured through time in serial microfluidic mass sensor arrays (Cermak et al., 2016). MAR-FISH provides single-cell activity measurements, but does not yield growth rates *per se*. Recent evidence suggests that two complementary techniques, SIP-nano-SIMS-FISH and SIP-Raman-FISH, hold the most promise for providing single-cell growth rates in both the lab and field experiments.

SIP-nano-SIMS-FISH has been used to estimate growth of anaerobic phototrophic bacteria as well as for cells in syntrophic and symbiotic partnerships (Musat et al., 2008; Orphan et al., 2009; Foster et al., 2011). However, the growth computations in these studies depended upon several variables with unspecified uncertainties, such as substrate pools' isotopic composition estimates, assumed assimilative/dissimilative relationships, and microscopic biovolume measurements used to derive elemental content based on allometry. Recognizing this limitation, Kopf et al. (2015) recently demonstrated that accurate single-cell growth rates could be derived by applying appropriate mathematical models to data obtained from bacterial chemostat cultures labeled with ^2^H_2_O under well-constrained conditions and analyzed by SIP-nano-SIMS.

Previous studies applying SIP-Raman to aquatic microbiology measured cell activity levels by altered Raman spectral features and then cataloged them either morphometrically or by combining with FISH (e.g., Huang et al., 2007b; Li et al., 2012; Berry et al., 2014). Huang et al. (2007b) were the first to demonstrate that red shift ratios in single-cell Raman spectra of cultivated bacteria varied predictably with ^13^C-content of the media. Li et al. (2012) recently demonstrated that SIP-SCRR red shifts in the three major carotenoid peaks ($\langle \Delta \tilde{\nu }\rangle$) vary quantitatively with *f*_cell_ of *Synechocystis* sp. and *Synechococcus elongatus*, grown in ^13^C-bicarbonate enriched media. In fact, when their data from these two cyanobacteria are combined, we obtain functional responses that closely agree with those presented in Figure 5; e.g., $\langle \Delta {\tilde{\nu }}_{C-C}\rangle$ = 1156.5–31.0±1.4 *f*_cell_; *r*^2^ = 0.99 (computed from Figure 3 in Li et al., 2012). The *f*_cell_ values derived from mass spectrometric analysis of extracted cellular protein (Li et al., 2012) yielded essentially the same functional responses as carotenoid $\langle \Delta \tilde{\nu }\rangle$. This is consistent with the postulates that turnover of major intracellular molecular pools are tightly coupled under optimal culture conditions and that the isotopic signatures are equivalent, i.e., α_protein_ ≈ α_carotenoids_, which supports the validity of our approach. Furthermore, it corroborates the proposition that as long as α and μ_pop_ are known and *f*_media_ is well-constrained, mean *f*_cell_ of an autotrophic culture can be reliably computed and precludes the need for mass spectrometry. To our knowledge, the present study is the first to extend SIP-SCRR data interpretation beyond fractional labeling and relative activity determinations to quantify absolute single-cell growth rates from photoautotrophic populations.

### Single-cell growth rates by SIP-SCRR-FISH

Our approach is well-suited to examine cell to cell heterogeneity in photoautotrophic growth in both cultures and field samples. Almost all carbon assimilated by aquatic photoautotrophs is drawn from the C_T_ pool. Therefore, dissolved ^13^C-bicarbonate, which equilibrates with C_T_ species, is an unsurpassed tracer of autotrophic production. Surface ocean values of C_T_ only vary between ~1.95 and 2.25 mM C globally (Key et al., 2004), and can be empirically measured in samples by several methods (e.g., Hall and Aller, 1992; Key et al., 2004; Kaltin et al., 2005). Therefore, the experimentalist can add precisely measured masses of H^13^${\text{CO}}_{3}^{-}$ to samples and accurately calculate *f*_media_, which is ultimately needed to derive μ_sc_. In contrast, DIC replacement manipulations lead to uncertainties in C_T_ and calculation of *f*_media_ (Table S1), necessarily alter water chemistry, and are impractical for field experiments. Therefore, we conclude that spiking unaltered samples with a sterile H^13^${\text{CO}}_{3}^{-}$ concentrate to achieve the desired *f*_media_ is preferable to any DIC replacement protocol (e.g., Li et al., 2012). In principle, our experiments demonstrated that H^13^${\text{CO}}_{3}^{-}$ amendments as small as 10% are adequate to exceed our LOD (3% Δ*f*_cell_) within 0.5 generations (Figure 5) and enable recognition of active cells. However, if accurate and precise determination of μ_sc_ is the objective, then H^13^${\text{CO}}_{3}^{-}$ amendments yielding 0.3–0.5 *f*_media_ and incubation durations of one generation are recommended to constrain the optimal propagated relative uncertainties (± σ_μ_/μ) to between 6.6 and 11.0% (see Figures S2–S4). These amendments do not appear to significantly alter photoautotrophic growth in seawater (Figure S1).

Sample processing requirements for SCRR or SCRR-FISH are relatively simple. Samples can be captured on membranes and frozen or preserved with formaldehyde or paraformaldehyde, but not glutaraldehyde (due to fluorescence interference). If FISH is required, samples are processed by standard membrane-based protocols (e.g., Pernthaler et al., 2001; Stoecker et al., 2010). In contrast, previous SIP-Raman-FISH studies required performing hybridizations on cell suspensions (liquid-FISH) concentrated by centrifugation or resuspension from a membrane, then spotting suspensions onto quartz or Al-coated glass slides, followed by drying, rinsing and DAPI counter-staining (Huang et al., 2007b; Berry et al., 2014). Membrane-based approaches are advantageous because cell recovery exceeds that obtained by centrifugation or by resuspension from membranes. Furthermore, the risk of cell loss from the microscope slide during rinsing or counter-staining in the liquid-FISH technique is eliminated.

Hybridization of fluorophore-labeled oligonucleotide FISH probes into cells may introduce two challenges to Raman interrogation; unwanted fluorescence and isotope dilution. Persistent fluorescence from a sample illuminated by a laser within its electronic transition band can prevent acquisition of Raman spectra altogether. As demonstrated by Huang et al. (2007b), bacterial cells hybridized with FISH probes labeled with Cy3, Cy5, or Fluorescein (Fluos) were all amenable to single-cell Raman microspectroscopy using a 532 nm laser, but cells labeled with Cy3 required brief photobleaching prior to acquisition of spectra. We only tested a Cy3-labeled probe using our 514 nm laser and could obtain high quality SCRR spectra of carotenoids without significant fluorescent interference. Nonetheless, Cy5, Fluos and perhaps other yet-to-be tested fluorophores are probably preferable to Cy3 for most applications, but practitioners should test fluorophores with desired laser line.

Isotope dilution created by incorporation of a FISH probe is a concern with SIP-nano-SIMS because the instrument is measuring secondary ions generated from individual atoms ablated from a specimen, irrespective of source molecules. Thus, ionized carbon from isotopically-enriched biomass can be diluted with ionized light carbon from a FISH probe. In the SIP-Raman-FISH approach, however, isotopic content determinations arise from wavenumber shifts in signature molecular bond vibrations, in the present case from ν(C-C) in carotenoids. Therefore, aliasing *f*_cell_ determinations from diagnostic SCRR peaks by isotopically-light FISH probes is improbable in the present study and can be avoided in other Raman assays. For example, Huang et al. (2007b) determined ^13^C-content in FISH-hybridized cells from red shift ratios in diagnostic peaks emanating from the essential amino acid, phenylalanine, were indistinguishable from unhybridized cells. Cells hybridized by CARD-FISH (catalyzed reporter deposition) may not be amenable to Raman interrogation because required reagents may be Raman-active and contaminate the spectra, but this requires future evaluation.

While membrane-based cell collection, hybridization, and staining techniques present significant advantages for working with microbes in natural waters, all tested membranes and filters are Raman-contaminating and consequently can't be used directly in the instrument. Therefore, we were motivated to develop a rapid, simple method to efficiently transfer concentrated and stained cells from low sorption membranes (polycarbonate) to a Raman-neutral substrate for interrogation. With minimal optimization efforts, the filter-transfer-freeze (FTF) technique met those goals and achieved transfer efficiencies of 51–77% for *Synechococcus* to mirror-finished stainless steel slides. For most anticipated applications, complete cell transfer is not required to adequately subsample populations captured on membranes. However, evaluation of other membrane materials may be warranted to maximize transfer efficiencies and minimize selective retention on membranes. The steel slides have several advantages. Firstly, they are completely reusable after solvent cleaning. Secondly, they cost 1–3% that of the quartz and Al-coated slides used in previous Raman studies (e.g., Huang et al., 2007b; Li et al., 2012). The third advantage is that potentially higher quantum efficiencies improve the method's sensitivity. This is explained as follows: Raman-shifted photons tend to scatter more or less in a 360° sphere (isotropic), depending on sample morphology, opacity, volume, and substrata properties. However, reflective opaque metal slides or Al-coated glass substrata limit scattering geometry to 180°, reflecting photons upward and thus potentially doubling photon capture efficiency compared to transparent substrata. Lastly, the highly reflective surface enables recognition of cells as small as bacteria under bright-field illumination without using any staining or FISH procedures.

### Intra-population growth rate heterogeneity

In this study, computed growth rates within a given isogenic *Synechococcus* sp. population varied from cell to cell by ~27% (CV) around the mean at any particular time point after removal of the ~2% propagated analytical error. Similar intra-population variations have been observed in isogenic populations of photoautotrophic protists. For example, microscopic cell count measurements of μ_sc_ for *Chlorella vulgaris* varied between 0.55 and 1.52 day^−1^ ($\bar{x}$ = 1.16 ± 0.37 SD) among mineral oil-encapsulated droplets of media within a microfluidic device, yielding a CV of 32%, while μ_pop_ in bulk media was 1.12 day^−1^ (Dewan et al., 2012). Similarly, subpopulations in a synchronized isogenic *Chlamydomonas reinhardtii* culture grew at significantly different rates among oil-encapsulated droplets within a millifluidic sampler (Damodaran et al., 2015). Using SIP-nano-SIMS, Kopf et al. (2015) reported CVs of 19–51% for μ_sc_ of chemostat cultures of the bacterium, *Staphylococcus aureus*. Interestingly, the heterogeneity in single cell growth rate (CV) varied inversely with chemostat dilution rate, i.e., behavior of individual cells in slow growing *S. aureus* populations was less uniform than that of fast growers. We observed a similar trend with fast-growing *T. pseudonana* (CV = 8–9%) and slower-growing *Synechococcus* sp. (CV = 23–34%) (Figure 8).

We are unaware of any comparable measurements of μ_sc_ in cyanobacteria. However, significant variations in elemental stoichiometry within *Synechococcus* field populations have been observed using synchrotron X-ray fluorescence microscopy. For example, an average CV of 120% in Si/P ratios was apparent among cells within discrete tropical and subtropical water samples (Baines et al., 2012). Similarly, quotas for P and Fe varied by 68 and 97% CV, respectively, among individual *Synechococcus* cells in the Sargasso Sea (Twining et al., 2010). Even individual *Synechococcus, Prochlorococcus*, and *Thalassiosira* cultures exhibit significant intra-population variability in intracellular P content (CV = 17–86%), which can affect the collective population growth rate (Fredrick et al., 2013). Thus, considerable cell-to-cell variation in elemental composition, activity, and growth of photoautotrophic populations can be expected whether they are isogenic and exposed to ostensibly homogenous conditions or are anisogenic and living in dynamic heterogeneous environments. Such findings and agent-based ecological models underscore the need for reliable single-cell measurements to better understand processes driving plankton population dynamics as well as biological cycling of key elements (Bolnick et al., 2011; Bucci et al., 2012; Fredrick et al., 2013).

## Conclusions

We conclude that, compared to other technologies, our approach to single-cell growth measurement has some clear advantages. SCRR has minimal sample preparation requirements, provides relatively high sample throughput, and at low cost per analysis (excluding capital expenses). Specimens can be interrogated *in vivo, in vitro*, frozen, preserved, or dried, and don't require special analytical conditions, such as electrical conductivity, embedding, or high vacuum (as in nano-SIMS). Organisms can be recognized by bright-field, autofluorescence, fluorescent stains, or FISH illumination. All targeted cells in a field can then be immediately analyzed by Raman microspectrometry by altering the optical path with a few computer keystrokes and allowing the automated stage to center each target under the laser spot. Raman microspectrometry offers a unique and powerful set of capabilities to chemically interrogate individual cells and investigate biogeochemical processes at spatial scales relevant to microorganisms. Combining SIP with FISH and Raman microspectrometry allows identification of key players in cycling of a particular element or compound and thereby enables linking function with phylogeny. Furthermore, rates of material cycling can be determined from features within single-cell Raman spectra. If the stable isotope traces the sole source of that element into cells and *f*_media_ of that element can be measured, then single-cell growth rates can be determined.

As demonstrated in this study, a ^13^C-bicarbonate tracer and SCRR of carotenoids appear to be particularly well-suited to investigate intra- and inter-population variability in growth of cultured photoautotrophs. As documented by Goericke and Welschmeyer (1993), carotenoid labeling rates closely match population growth rates during balanced growth which occurs during exponential phase in cultures. However, they also observed that nutrient and light stresses may alter this relationship, conditions which may be encountered in field samples. We recommend conducting SIP experiments over an entire diel cycle (24 h) to account for variability in light field responses and intrinsic biosynthetic periodicities and to produce sufficient signal. Resolution of the impact of light and nutrient limitation on broad application of the SIP-SCRR approach to examining intra- and inter-population growth variability in the field requires further systematic evaluation under a range of conditions.

## Author contributions

All authors listed have made a substantial, direct and intellectual contribution to the work, and approved it for publication.

### Conflict of interest statement

The authors declare that the research was conducted in the absence of any commercial or financial relationships that could be construed as a potential conflict of interest.
