# Supplementary material for: Single-Cell Growth Rates in Photoautotrophic Populations Measured by Stable Isotope Probing and Resonance Raman Microspectrometry
Source: Front Microbiol. 2017 Aug 3;8:1449. doi: 10.3389/fmicb.2017.01449 (PMC5541042; doi:10.3389/fmicb.2017.01449)
Supplement: Supplementary file 1 [file Presentation1.pdf]

Supplementary Material

Single-Cell Growth Rates in Photoautotrophic Populations Measured by Stable Isotope Probing and Resonance Raman Microspectrometry

Gordon T. Taylor*[1], Elizabeth Suter[1], Zhuo Qun Li[1], Stephanie Chow[1], Dallyce Stinton[1], Tatiana Zaliznyak[1], and Steven R. Beaupré[1]

1. School of Marine and Atmospheric Sciences, Stony Brook University, Stony Brook, NY, USA

*** Correspondence:** Gordon T. Taylor: gordon.taylor@stonybrook.edu

SM1: Evaluation of ^{13}C-enriched media preparation methods

The first critical steps in performing quantitative SIP experiments with cultures is to establish whether DIC replacement or augmentation is preferable, to determine how much ^{13}C-bicarbonate tracer can be added without significantly altering seawater chemistry, and then to accurately determine the fractional contribution ($f_{media} = {}^{13}C_{media} / ({}^{13}C_{media} + {}^{12}C_{media})$) of the heavy isotope to the total inorganic carbon pool ($C_T = CO_2 + H_2CO_3 + HCO_3^- + CO_3^{2-}$).

Three DIC replacement methods were evaluated for how effectively DI^{12}C was removed prior to media preparation and ^{13}C-bicarbonate amendment. The first method was boiling filtered seawater (FSW) for 1 min in a microwave oven according to Li et al. (2012). The second was to actively sparge FSW with N_2 for 10 min. The third approach was to acidify FSW to pH 3.5 with 1N HCl, then seal in a gas-tight vessel and autoclave. Once cool, pH was returned to 8.0 with 0.1N NaOH. Standard f/2 nutrients and sodium bicarbonate were aseptically transferred to 200-ml septum bottles to yield a final C_T of ~2 mM in nominal f_{media} ratios of 0.011 (natural abundance), 0.25, 0.50, 0.75, and 1.00. However, media prepared in this way usually became viscous and turbid due to mineral precipitation and *Synechococcus* sp. cultures did not grow well under these conditions.

DI^{13}C augmentation to the culture media was evaluated for pH and growth effects. At the extreme, 2.3 mM DI^{13}C additions (final C_T ~4.1 mM or 130% enrichment) only depressed pH by 0.23 units (Table S1). At C_T enrichments of <50%, pH excursions were < 0.13, suggesting that amendments at these levels will not significantly affect photoautotrophic growth.

Total dissolved inorganic carbon (C_T) concentrations were determined using a flow injection analysis (FIA) system (Hall and Aller, 1992). This instrument permits transfer of CO_2 from an acidified (ΣCO_2) reagent stream across a gas-permeable membrane into a receiving carrier stream which flows over a conductivity detector. C_T levels of unknown samples were compared to conductivity measurements of known standards. pH measurements were made on a Thermo

Scientific® Orion 2-Star™ Benchtop pH meter, which was calibrated immediately prior to all measurements.

In principle, for accurate $DI^{13}C$ replacement, all or a known amount of the C_T pool must be removed before replacing with sufficient ^{13}C-bicarbonate to return media to the original C_T pool size. Microwave heating as employed by Li et al. (2012) or N_2 sparging only removed 30 or 60% of the original C_T pool, respectively, while acidification effectively removed 96% of the C_T pool as determined by FIA-conductivity measurements of ΣCO_2 (Table S2). Microwave heating had the additional effect of raising the pH above 9.0. Adding 1.8 mmol L^{-1} bicarbonate to previously acidified and neutralized FSW returned DIC to the original pool size and an acceptable pH. Doing the same to the microwaved or sparged samples in the present case would have increased C_T by 71 and 40% and without independent verification, the actual f_{media} would be poorly constrained. Therefore, pH manipulation is clearly the most reliable approach for DIC replacement.

Partial or complete DIC replacement may be a suitable approach for media preparation and culture experiments, but clearly is inappropriate for field experiments with natural assemblages. DIC augmentation requires far less manipulation and therefore introduces fewer potential artifacts. Alteration of pH is foremost among potential artifacts for DIC augmentation and was therefore evaluated. We note that pH of the aged seawater (Table S1) was higher than that of average seawater (pH = 7.5-8.4), which we attribute to prolonged photoautotrophic growth within the storage vessel. Increasing C_T by 11% to as much as 130% depressed the pH of FSW from 8.79 to 8.56 (Table S1). Consequently, our experimental data were derived from media augmented with a constant amount of total DIC, but varying ^{13}C-bicarbonate content, and then returned to a pH of 8.0. Reported f_{media} are based on IRMS and FIA-conductivity results, which were within ~6% of nominal ratios (gravimetrically formulated from C_T concentration in FSW) (Table S2).

Table S1. Summary of effects of dissolved inorganic carbon manipulations on cultivation medium.

**Treatment**	**C_T[f]** (mean mM ± SD)	**pH**
Filtered coastal seawater (FSW)	1.78 ± 0.10	8.76
Microwaved to boiling FSW [a]	1.25 ± 0.06	9.04
N_2-purged FSW [b]	0.72 ± 0.08	8.30
Acidified FSW [c]	0.07 ± 0.07	3.50
Previously acidified FSW, pH adjusted & bicarbonate added [d]	1.86 ± 0.06	8.00
FSW – 11% DIC enriched [e]	1.98	8.79
FSW – 22% DIC enriched [e]	2.16	8.76
FSW – 51% DIC enriched [e]	2.69	8.66
FSW – 77% DIC enriched [e]	3.16	8.57
FSW – 130% DIC enriched [e]	4.09	8.56

a – FSW brought to a boil in microwave and held for 1 min according to Li et al. (2012).

b – FSW sparged for 10 min with N_2 for 10 ml FSW – sparged for 30 min for 200 ml

c – FSW acidified with 1.0 N HCl, shaken in a closed vial, and opened to release $^{12}CO_2$ gas

d – pH of FSW from c was adjusted with 0.1 N NaOH.

e – Unmanipulated FSW was amended with increasing volumes of a 10 mM $NaHCO_3$ working stock. pH was returned to 8.0 prior to inoculation with Synechococcus cells.

f – $C_T = CO_2 + CO_3^{-2} + HCO_3^{-}$ and determined as ΣCO_2 by flow injection and conductivity analysis of acidified sample (Hall and Aller, 1992)

Table S2. Comparison of C_T values derived from three methods for SIP experiments using 2 mM DIC amendments with varying f_{media}.

**Method**	**Mean** (mM)	**S.D.**	**N**	**Significantly Different** [a]
Gravimetric (G)	3.78	na[b]	1	from IRMS
Flow Injection Analysis (FIA)	3.78	0.10	8	from IRMS
Isotope Ratio Mass Spectrometry (IRMS)	3.56	0.05	12	from G and FIA

a – p < 0.05; Dunn's Method pairwise ANOVA
b – not applicable because concentration based on a single measurement

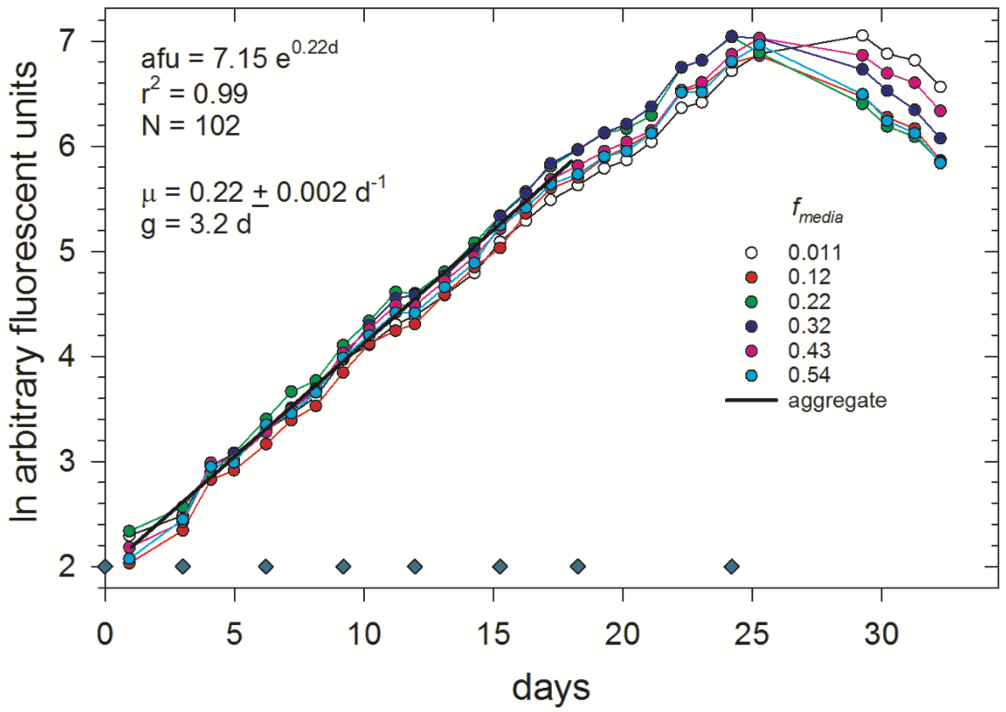

Figure S1. Growth curves determined by *in vivo* fluorescence (460 nm excitation / >665 nm emission) for *Synechococcus* sp. (RS9916) grown in normal f/2 media (f_{media} = 0.011, control) and f/2 media augmented with 2 mM ^{13}C-bicarbonate to yield f_{media} = of 0.12, 0.22, 0.32, 0.43, and 0.54. Mean growth rates (μ_{pop}) determined by slopes over first 18 days were not significantly different among treatments (ANOVA $p > 0.90$). Aggregate curve and regression include data from all six cultures. Diamonds indicate dates on which samples were collected for Raman microspectrometry.

SM2: Expected relationships between mean SIP-SCRR wavenumbers ($\langle\Delta\tilde{\nu}\rangle$) and fractional isotopic abundance (f_{cell})

Slight differences in vibrational frequencies upon substituting ^{13}C for ^{12}C in a carbon-carbon bond are manifested as unique peaks in the Raman spectrum. Since these isotopologue peaks tend to overlap, their *relative areas* are imprecise predictors of the fractional isotopic abundance of ^{13}C (f_{13}). However, a strong relationship exists between the *mean Raman wavenumber* of overlapping isotopologue peaks and fractional isotopic abundance, which can be derived as follows.

First, we assume that all isotopologue bonds have symmetric Raman peaks with characteristic central wavenumbers ($\Delta\tilde{\nu}_i$, where the subscript $i = 0, 1, 2$ is the number of ^{13}C atoms in $^{12}C^{12}C$, $^{12}C^{13}C$, or $^{13}C^{13}C$ bonds, respectively). Therefore, the mean wavenumber ($\langle\Delta\tilde{\nu}\rangle$) for overlapping isotopologue bond peaks is the average of their characteristic wavenumbers weighted by their peak areas (A_i).

(S1) $$\langle\Delta\tilde{\nu}\rangle = \frac{A_0\Delta\tilde{\nu}_0 + A_1\Delta\tilde{\nu}_1 + A_2\Delta\tilde{\nu}_2}{A_0 + A_1 + A_2}$$

The areas are obtained by integrating each peak over all values of $\Delta\tilde{\nu}$. We assume that the peak heights are equal to the products of their Raman efficiencies ($\varepsilon_i(\Delta\tilde{\nu})$, the effective wavenumber-dependent cross-section with units of intensity · effective cell area · $bond_i^{-1}$) and the number of isotopologue bonds of interest in the optical path. The latter is equal to the product of the average cell thickness (l), concentration of the molecule of interest in the cell ([m]), stoichiometric number of bonds in that molecule expressing the desired resonance (e.g., $b = 9$ conjugated double bonds in the chain per β-carotene), and expected proportions of isotopologues among those bonds ($\langle\phi_i\rangle$). These terms are constants on the timescale of acquiring a Raman spectrum can be factored out of the peak integral. Therefore, the peak area (intensity · cm^{-1}) is the product of these constants and a bulk efficiency (ε_i', with units of intensity · effective cell area · $bond_i^{-1}$ · cm^{-1}) that is equal to the integral of $\varepsilon_i(\Delta\tilde{\nu})$ over all values of $\Delta\tilde{\nu}$ (Eq. S2).

(S2) $$A_i = \varepsilon_i' l[m]b\langle\phi_i\rangle$$

The expected proportions of $^{12}C^{12}C$, $^{12}C^{13}C$, and $^{13}C^{13}C$ bonds can be estimated by assuming a stochastic distribution of ^{13}C atoms throughout the principle resonance structure of the molecule (i.e., assuming negligible isotopic clumping). Hence, the *proportions* (probability = $P(k|n,f_{13})$) of each set of isotopomers with k ^{13}C atoms in a molecule containing n carbon atoms will be given by the individual terms of the binomial distribution (Eq. S3), where the bulk binomial coefficient ("n over k" term) represents the number of possible unique isotopomers in that set.

(S3) $$P(k|n, f_{13}) = \binom{n}{k} f_{13}^k (1 - f_{13})^{n-k}$$

As such, any molecule with n carbon atoms for which $k = 0$ to n ^{13}C atoms are randomly substituted could exist as 2^n possible isotopic species. For example, β-carotene ($C_{40}H_{56}$) has $2^{40} \approx$ 1.1 trillion possible isotopologues with respect to stable carbon isotopes.

(S4)
$$\sum_{k=0}^{n} \binom{n}{k} = 2^n$$

Of this total, the sum of all combinations of unique isotopologue bonds ($\eta_{i,k}$) *in each set of isotopomers defined by k* must be equal to the product of the number of all possible isotopologues and the number of bonds of interest in that molecule.

(S5)
$$\sum_{i=0}^{2} \eta_{i,k} = b\binom{n}{k}$$

Thus, expressions for the number of possible $^{12}C^{12}C$, $^{12}C^{13}C$, and $^{13}C^{13}C$ *bonds in each set of isotopomers defined by k* can be obtained by factoring combinations out of the bulk binomial coefficient that are consistent with the physical limitations of isotopic substitution (Eq. S6). Specifically, n is decreased by the number of atoms in a bond (i.e., 2) and k is decreased by i ^{13}C atoms because, for example, there cannot be any $^{13}C^{13}C$ bonds in a molecule with just $k = 0$ or 1 ^{13}C atoms, nor can there be any $^{12}C^{12}C$ bonds in a molecule with at least n – 1 ^{13}C atoms. Accordingly, each combination is only meaningful for values of k that range from i to $n - 2 + i$. The additional binomial coefficient ("2 over i") accounts for two possible sites of ^{13}C substitution in a $^{12}C^{13}C$ bond ($^{12}C^{13}C$ vs. $^{13}C^{12}C$), while the coefficient b scales the result to account for all possible bonds of interest in the set.

(S6)
$$\eta_{i,k} = \begin{cases} b\binom{2}{i}\binom{n-2}{k-i}, & for\ i \le k \le n-2+i \\ 0, & otherwise \end{cases}$$

Therefore, the proportions ($\phi_{i,k}$) of the numbers of possible $^{12}C^{12}C$, $^{12}C^{13}C$, and $^{13}C^{13}C$ bonds in each set of isotopologues defined by k are found by dividing Eq. S6 by Eq. S5.

(S7)
$$\phi_{i,k} = \begin{cases} \binom{n}{k}^{-1}\binom{2}{i}\binom{n-2}{k-i}, & for\ i \le k \le n-2+i \\ 0, & otherwise \end{cases}$$

The expected proportions of $^{12}C^{12}C$, $^{12}C^{13}C$, and $^{13}C^{13}C$ bonds in the entire suite of 2^n isotopic species are equal to the averages of their proportions in each set defined by k.

(S8)
$$\langle \phi_i \rangle = \sum_{k=i}^{n-2+i} P(k|n, f_{13}) \phi_{i,k}$$

Substituting Eq. S3 and S7 into S8 gives an expression for the expected proportions of $^{12}C^{12}C$, $^{12}C^{13}C$, and $^{13}C^{13}C$ bonds as a function of f_{13}, n, and k.

(S9)
$$\langle \phi_i \rangle = \sum_{k=i}^{n-2+i} \binom{2}{i} \binom{n-2}{k-i} f_{13}^{k} (1 - f_{13})^{n-k}$$

These expected proportions can be greatly simplified by first implementing a change of variables and factoring (let $n' = n - 2$, and $k' = k - i$).

(S10)
$$\langle \phi_i \rangle = \binom{2}{i} f_{13}^{i} (1 - f_{13})^{2-i} \sum_{k'=0}^{n'} \binom{n'}{k'} f_{13}^{k'} (1 - f_{13})^{n'-k'}$$

The summation in Eq. S10 represents a complete binomial distribution and is therefore equal to one. Thus, the expected stochastic proportions of $^{12}C^{12}C$, $^{12}C^{13}C$, and $^{13}C^{13}C$ bonds are independent of the number of atoms or bonds of interest in a molecule (where $n \geq 2$). Instead, they are equivalent to the proportions expected for a diatomic molecule via binomial expansion.

(S11)
$$\langle \phi_i \rangle = \binom{2}{i} f_{13}^{i} (1 - f_{13})^{2-i}$$

or, after expanding the terms:

$$\langle \phi_0 \rangle = (1 - f_{13})^2$$
$$\langle \phi_1 \rangle = 2 f_{13} (1 - f_{13})$$
$$\langle \phi_2 \rangle = f_{13}^2$$

Finally, substituting Eq. S2 and S11 into Eq. S1 and simplifying yields the expected general relationship between mean SCRR shift and fractional ^{13}C abundance.

(S12)
$$\langle \Delta\tilde{\nu} \rangle = \frac{\varepsilon_0' \Delta\tilde{\nu}_0 + 2(\varepsilon_1' \Delta\tilde{\nu}_1 - \varepsilon_0' \Delta\tilde{\nu}_0) f_{13} + (\varepsilon_2' \Delta\tilde{\nu}_2 - 2\varepsilon_1' \Delta\tilde{\nu}_1 + \varepsilon_0' \Delta\tilde{\nu}_0) f_{13}^2}{\varepsilon_0' + 2(\varepsilon_1' - \varepsilon_0') f_{13} + (\varepsilon_2' - 2\varepsilon_1' + \varepsilon_0') f_{13}^2}$$

This expression is consistent with our intuition that the mean Raman shift ($\langle \Delta\tilde{\nu} \rangle$) is equal to the characteristic wavenumber of a $^{12}C^{12}C$ resonance ($\Delta\tilde{\nu}_0$) when $f_{13} = 0$, or a $^{13}C^{13}C$ resonance ($\Delta\tilde{\nu}_2$)

when $f_{13} = 1$, and it is symmetrically weighted around the resonance of a $^{12}C^{13}C$ bond ($\Delta\tilde{\nu}_1$) when $f_{13} = 0.5$. This function is linear when assuming approximately equal efficiencies ($\varepsilon'_0 \approx \varepsilon'_1 \approx \varepsilon'_2$) and spacing between peaks (i.e., $\Delta\tilde{\nu}_0 - \Delta\tilde{\nu}_1 \approx \Delta\tilde{\nu}_1 - \Delta\tilde{\nu}_2$). If we define f_{cell} as the f_{13} value of a specific compound in a cell, then $\langle\Delta\tilde{\nu}\rangle$ can be expressed as a linear function of f_{cell} with an intercept and a slope predicted from theory ($\Delta\tilde{\nu}_0$ and $2(\Delta\tilde{\nu}_1 - \Delta\tilde{\nu}_0)$; Eq. S13) or obtained by experiment (b_0 and b_1; Eq. S14).

(S13)
$$\langle\Delta\tilde{\nu}\rangle \approx \Delta\tilde{\nu}_0 + 2(\Delta\tilde{\nu}_1 - \Delta\tilde{\nu}_0)f_{cell}$$

or

(S14)
$$\langle\Delta\tilde{\nu}\rangle \approx b_0 + b_1 f_{cell}$$

This line of reasoning predicts that the observed intercept (b_0) is equal to the $^{12}C^{12}C$ resonance ($\Delta\tilde{\nu}_0$), and that the slope (b_1) is both negative (because $\Delta\tilde{\nu}_2 < \Delta\tilde{\nu}_1 < \Delta\tilde{\nu}_0$) and approximately equal in magnitude to twice the spacing between adjacent peaks. This model is supported by the observed linearity between $\langle\Delta\tilde{\nu}\rangle$ and f_{cell} ($r^2 = 0.96$), and by agreement between the values of $\Delta\tilde{\nu}_1$ and $\Delta\tilde{\nu}_2$ that are observed (1498 cm^{-1}, 1474 cm^{-1}) and expected ($\Delta\tilde{\nu}_{1,expected} = b_0 + b_1/2 =$ 1498.7 ± 0.3 cm^{-1}, $\Delta\tilde{\nu}_{2,expected} = b_0 + b_1 =$ 1475.4 ± 0.3 cm^{-1}) from regression parameters ($b_1 = -44.9 \pm 0.30$, and $b_0 = 1521.8 \pm 0.08$ cm^{-1}) for β-carotene's ν(C=C) stretch in *Synechococcus* cells. However, the model should be applicable to any resonance that meets the stated assumptions.

SM3: Expected relationship between fractional isotopic abundance (f_{cell}), generation number (n), and mean SIP-SCRR wavenumbers ($\langle \Delta\tilde{\nu} \rangle$)

The relationship between f_{cell} and the number of generations (j, where $0 \leq j \leq n$) can be estimated assuming that (1) shifts in fractional isotopic abundances due to Rayleigh distillations are negligible on the timescale of a typical incubation, (2) variations in the isotopic fractionation factor (α) associated with biosynthesis of carotenoids are within error of α for bulk cellular production, (3) all cells in the population have approximately the same concentration of carbon atoms (moles/cell), (4) all cellular constituents turn over on approximately the same timescale, and (5) that ancestral and newly assimilated biomass are isotopically homogenized prior to cell fission. Under these assumptions, the number of cells (N) in the original inoculum (subscript 0) doubles with each new generation (j).

(S15) $$N_j = N_o e^{j ln(2)}$$

According to isotopic mass balance, the fractional isotopic abundances of daughter cells ($f_{\mathrm{cell},j+1}$) will be the simple average of the isotopic abundances of their parent cells ($f_{\mathrm{cell},j}$) and of the ^{13}C-enriched biomass that was assimilated prior to fission (f_{new}).

(S16) $$f_{cell,j+1} = \left(f_{cell,j} + f_{new}\right)\frac{N_j}{N_{j+1}}$$

Recursive application of Eq. S16 and simplification permits calculation of the fractional isotopic abundance of the $j = n^{\mathrm{th}}$ generation of cells from the number (N_{o}) and fractional isotopic abundance ($f_{\mathrm{cell,o}}$) of the original generation of cells ($j = 0$).

(S17) $$f_{cell,n} = f_{cell,o}\frac{N_o}{N_n} + f_{new}\frac{1}{N_n}\sum_{j=0}^{n-1} N_j$$

Substituting Eq. S15 into Eq. S17 reduces the summation to $N_n - N_o$ and the ensuing $N_{\mathrm{o}}/N_{\mathrm{n}}$ terms to $e^{-n\,\ln(2)}$, producing a general isotopic mass balance expression for f_{cell} after n generations.

(S18) $$f_{cell,n} = f_{new} + \left(f_{cell,o} - f_{new}\right)e^{-n ln(2)}$$

When constructing a calibration curve ($\langle \Delta\tilde{\nu} \rangle$ vs. f_{cell}) it is easier to directly measure the fractional isotopic abundances of growth media (f_{media}) than of discrete cellular constituents. Therefore, the definitions for fractionation factors ($\alpha_{\mathrm{biomass\text{-}media}} = R_{\mathrm{biomass}}/R_{\mathrm{media}}$), isotope ratios ($R = {}^{13}\mathrm{C}/{}^{12}\mathrm{C}$), and fractional isotopic abundances ($f_{13} = {}^{13}\mathrm{C}/({}^{12}\mathrm{C}+{}^{13}\mathrm{C})$) can be used to predict the isotopic composition of cellular biomass from the isotopic composition of growth media.

(S19) $$f_{biomass} = \frac{\alpha f_{media}}{1 + (\alpha - 1) f_{media}}$$

Substituting Eq. S19 for f_{new} and $f_{cell,o}$ in Eq. S18 gives the following expressions for calculating f_{cell} and its uncertainty (σ_{fcell}), where f_o and f_{media} are the fractional isotopic abundances of the original and ^{13}C-enriched growth media, respectively.

(S20) $$f_{cell} = \frac{\alpha f_{media}}{1 + (\alpha - 1) f_{media}} + \left(\frac{\alpha f_o}{1 + (\alpha - 1) f_o} - \frac{\alpha f_{media}}{1 + (\alpha - 1) f_{media}} \right) e^{-nln(2)}$$

(S21) $$\sigma_{f_{cell}} = \sqrt{\left(\frac{\partial f_{cell}}{\partial f_o}\right)^2 \sigma_{f_o}^2 + \left(\frac{\partial f_{cell}}{\partial f_{media}}\right)^2 \sigma_{f_{media}}^2 + \left(\frac{\partial f_{cell}}{\partial n}\right)^2 \sigma_n^2 + \left(\frac{\partial f_{cell}}{\partial \alpha}\right)^2 \sigma_\alpha^2}$$

where

$$\left(\frac{\partial f_{cell}}{\partial f_o}\right) = \frac{\alpha}{(1 + (\alpha - 1) f_o)^2} e^{-nln(2)}$$

$$\left(\frac{\partial f_{cell}}{\partial f_{media}}\right) = \frac{\alpha}{(1 + (\alpha - 1) f_{media})^2} \left(1 - e^{-nln(2)}\right)$$

$$\left(\frac{\partial f_{cell}}{\partial n}\right) = \frac{\alpha (f_{media} - f_o) ln(2)}{(1 + (\alpha - 1) f_{media})(1 + (\alpha - 1) f_o)} e^{-nln(2)}$$

$$\left(\frac{\partial f_{cell}}{\partial \alpha}\right) = \frac{(1 - f_{media}) f_{media}}{(1 + (\alpha - 1) f_{media})^2} \left(1 - e^{-nln(2)}\right) + \frac{(1 - f_o) f_o}{(1 + (\alpha - 1) f_o)^2} e^{-nln(2)}$$

Substituting Eq. S20 for f_{cell} in Eq. S14 yields the expected relationship between $\langle \Delta\tilde{v} \rangle$ and n where, again, b_0 and b_1 are experimentally determined coefficients that are theoretically equal to $\Delta\tilde{v}_0$ and $2(\Delta\tilde{v}_1 - \Delta\tilde{v}_0)$, respectively.

(S22) $$\langle \Delta\tilde{v} \rangle \approx b_0 + b_1 \left(\frac{\alpha f_{media}}{1 + (\alpha - 1) f_{media}} + \left(\frac{\alpha f_o}{1 + (\alpha - 1) f_o} - \frac{\alpha f_{media}}{1 + (\alpha - 1) f_{media}} \right) e^{-nln(2)} \right)$$

SM4: Calculating the number of generations (n) from SCRR ⟨Δṽ⟩ measurements, associated uncertainties, and experimental design

The general equation for calculating the number of generations (n) from f_{media}, f_o, α, b_0, and b_1 is obtained by rearranging Eq. S22.

(S23) $$n \approx \frac{1}{ln(2)} ln\left(\frac{f_{media} - f_o}{(1 + (\alpha - 1)f_o)\left(f_{media} - (1 + (\alpha - 1)f_{media})\frac{\langle\Delta\tilde{\nu}\rangle - b_0}{\alpha b_1}\right)}\right)$$

Ignoring the $(1+(\alpha-1)f_o)$ term in the denominator of the logarithm will introduce a constant but negligible offset of +0.0004 generations for $\alpha = 0.976$ and $f_o = 0.011$. Therefore, n can be calculated more simply via Eq. S24.

(S24) $$n \approx \frac{1}{ln(2)} ln\left(\frac{f_{media} - f_o}{f_{media} - (1 + (\alpha - 1)f_{media})\frac{\langle\Delta\tilde{\nu}\rangle - b_0}{\alpha b_1}}\right)$$

The anticipated uncertainty on n and the experimental conditions needed to minimize this uncertainty, can be evaluated by propagating the uncertainties (single standard deviations, σ_i) on all terms in Eq. S24.

(S25) $$\sigma_n = \sqrt{\left(\frac{\partial n}{\partial f_o}\right)^2 \sigma_{f_o}^2 + \left(\frac{\partial n}{\partial f_{media}}\right)^2 \sigma_{f_{media}}^2 + \left(\frac{\partial n}{\partial b_1}\right)^2 \sigma_{b_1}^2 + \left(\frac{\partial n}{\partial b_0}\right)^2 \sigma_{b_0}^2 + \left(\frac{\partial n}{\partial \langle\Delta\tilde{\nu}\rangle}\right)^2 \sigma_{\langle\Delta\tilde{\nu}\rangle}^2 + \left(\frac{\partial n}{\partial \alpha}\right)^2 \sigma_{\alpha}^2}$$

where

$$\left(\frac{\partial n}{\partial f_o}\right) = \frac{-1}{(f_{media} - f_o) ln(2)}$$

$$\left(\frac{\partial n}{\partial f_{media}}\right) = \frac{1}{(f_{media} - f_o) ln(2)}\left(\frac{f_o - (1 + (\alpha - 1)f_o)\frac{\langle\Delta\tilde{\nu}\rangle - b_0}{\alpha b_1}}{f_{media} - (1 + (\alpha - 1)f_{media})\frac{\langle\Delta\tilde{\nu}\rangle - b_0}{\alpha b_1}}\right)$$

$$\left(\frac{\partial n}{\partial b_1}\right) = \frac{-1}{b_1 ln(2)}\left(\frac{(1 + (\alpha - 1)f_{media})\frac{\langle\Delta\tilde{\nu}\rangle - b_0}{\alpha b_1}}{f_{media} - (1 + (\alpha - 1)f_{media})\frac{\langle\Delta\tilde{\nu}\rangle - b_0}{\alpha b_1}}\right)$$

$$\left(\frac{\partial n}{\partial b_o}\right) = \frac{-1}{\alpha b_1 ln(2)}\left(\frac{1 + (\alpha - 1)f_{media}}{f_{media} - (1 + (\alpha - 1)f_{media})\frac{\langle\Delta\tilde{\nu}\rangle - b_0}{\alpha b_1}}\right)$$

$$\left(\frac{\partial n}{\partial\langle\Delta\tilde{\nu}\rangle}\right) = \frac{1}{\alpha b_1 ln(2)}\left(\frac{1+(\alpha-1)f_{media}}{f_{media}-(1+(\alpha-1)f_{media})\frac{\langle\Delta\tilde{\nu}\rangle-b_0}{\alpha b_1}}\right)$$

$$\left(\frac{\partial n}{\partial\alpha}\right) = \frac{1}{\alpha ln(2)}\left(\frac{(f_{media}-1)\frac{\langle\Delta\tilde{\nu}\rangle-b_0}{\alpha b_1}}{f_{media}-(1+(\alpha-1)f_{media})\frac{\langle\Delta\tilde{\nu}\rangle-b_0}{\alpha b_1}}\right)$$

Finally, the relative uncertainty of SIP-SCRR generation times ($g = t/n$) and specific growth rates ($\mu_{sc} = \ln(2)\ n/t$) can be estimated through propagation of uncertainties and the reasonable assumption that $\sigma_t/t \ll \sigma_n/n$.

(S26) $$\frac{\sigma_{\mu_{sc}}}{\mu_{sc}} = \frac{\sigma_g}{g} = \frac{\sigma_n}{n}$$

The values of most parameters in Eq. S24 and S25 are beyond the analyst's control (α, b_1, b_0, $\langle\Delta\tilde{\nu}\rangle$, f_o). However, an experiment can be optimized by careful selection of the ^{13}C-enriched growth medium (f_{media}) and the duration of an incubation (i.e., t or n). First, the most precise measurements of n and the growth rate must happen after shifts in f_{cell} become detectable ($n > 0$) but before f_{cell} reaches its asymptote (i.e., $f_{cell} = \alpha f_{media}/(1+(\alpha-1)f_{media})$, as per Eq. S20) (Figs. S2 and S3). This optimum condition was readily found for the ν(C-C) stretch by numerically locating the minimum value of the relative uncertainty ($\pm\,\sigma_n/n$) as a function of $\langle\Delta\tilde{\nu}\rangle$ for various values of f_{media} (ranging from 0 to 1, ±0.002). This required assuming reasonable values and uncertainties for α (0.976 ± 0.003), f_o (0.0110 ± 0.0002), and $\langle\Delta\tilde{\nu}\rangle$ (ranging from 1120 to 1160 cm^{-1}, with $\sigma_{\langle\Delta\tilde{\nu}\rangle} = \pm\, 0.34$) based on known measurements. The two remaining quantities, b_1 (-30.34 ± 0.18 cm^{-1}) and b_0 (1157.04 ± 0.05 cm^{-1}), were obtained from a least squares linear regression of 961 individual measurements ($r^2 = 0.97$; Fig. S2).

This analysis suggests that the most precise determinations of n will be obtained by measuring $\langle\Delta\tilde{\nu}\rangle$ after ~1.5 cell divisions. More importantly, the minimum relative uncertainties ($\pm 1\sigma_n/n$) can be significantly reduced from 0.356 ($f_{media} = 0.10$) to a theoretically lowest achievable value of 0.033 (i.e., in the limit of $f_{media} = 1.0$) by using more isotopically-enriched growth media (Fig. S2). In addition, cells grown in highly enriched media exhibit both broader and flatter relative uncertainty minima (Fig. S2), making the highest precision measurements much less sensitive to the number of generations or the precise timing of measurements. As a practical compromise between measurement performance, incubation artifacts, and costs, we advocate amending routine samples to f_{media} values between 0.3 and 0.5, where the optimum value of n has a minimum theoretical relative uncertainty between 0.11 and 0.066, respectively (Fig. S3).

Further analysis of Eq. S25 suggests that the greatest contribution of uncertainty (i.e., the individual "$(\partial n/\partial x)^2\sigma_x^2$" terms under the radical, where x = α, f_o, f_{media}, b_0, b_1, or $\langle\Delta\tilde{\nu}\rangle$) to SCRR determinations of n comes from measurements of $\langle\Delta\tilde{\nu}\rangle$ (Fig S4). Under optimal measurement conditions, the uncertainty contributions from measurements of α and f_o are at least 3 to 5 orders of magnitude smaller than that of $\langle\Delta\tilde{\nu}\rangle$, respectively, and therefore can be ignored. The remaining

measurements (f_{media}, b_0, and b_1) make approximately equal contributions to the total uncertainty and suggest avenues for future improvements in performance. Therefore, the accuracy and precision of SIP-SCRR growth rate determinations are presently limited by individual $\langle \Delta\tilde{v} \rangle$ measurements and the calibration curve (b_0 and b_1), rather than uncertainty or variations in the isotopic composition of original growth medium (f_o) or fractionation factors for carotenoid biosynthesis (α).

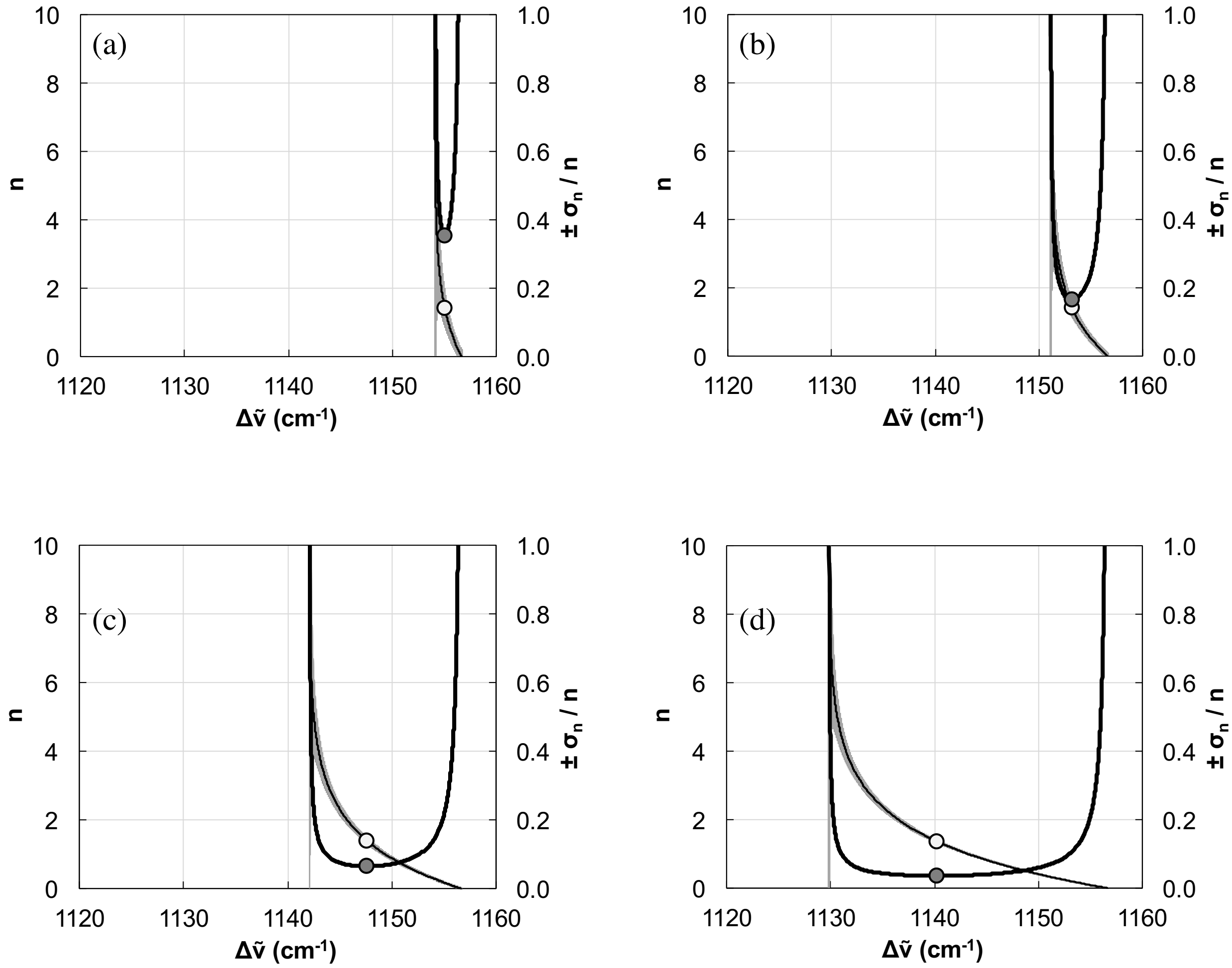

Figure S2. Modeled number of generations (n, thin black line), their uncertainties (± $1\sigma_n$, gray shaded area), and their relative uncertainties (σ_n/n, heavy black line) as a function of measured mean SCRR ν(C-C) shift for f_{media} values of (a) 0.10, (b) 0.20, (c) 0.50, and (d) 0.90, assuming α = 0.976 ± 0.003, b_1 = -30.34 ± 0.18 cm^{-1}, b_0 = 1157.04 ± 0.05 cm^{-1}, f_o = 0.0110 ± 0.0002, and $\sigma_{\nu(C-C)}$ = 0.34 cm^{-1}. Minimum values of $\pm\sigma_n/n$ and corresponding (optimal) values of n are indicated by the filled and open circles, respectively.

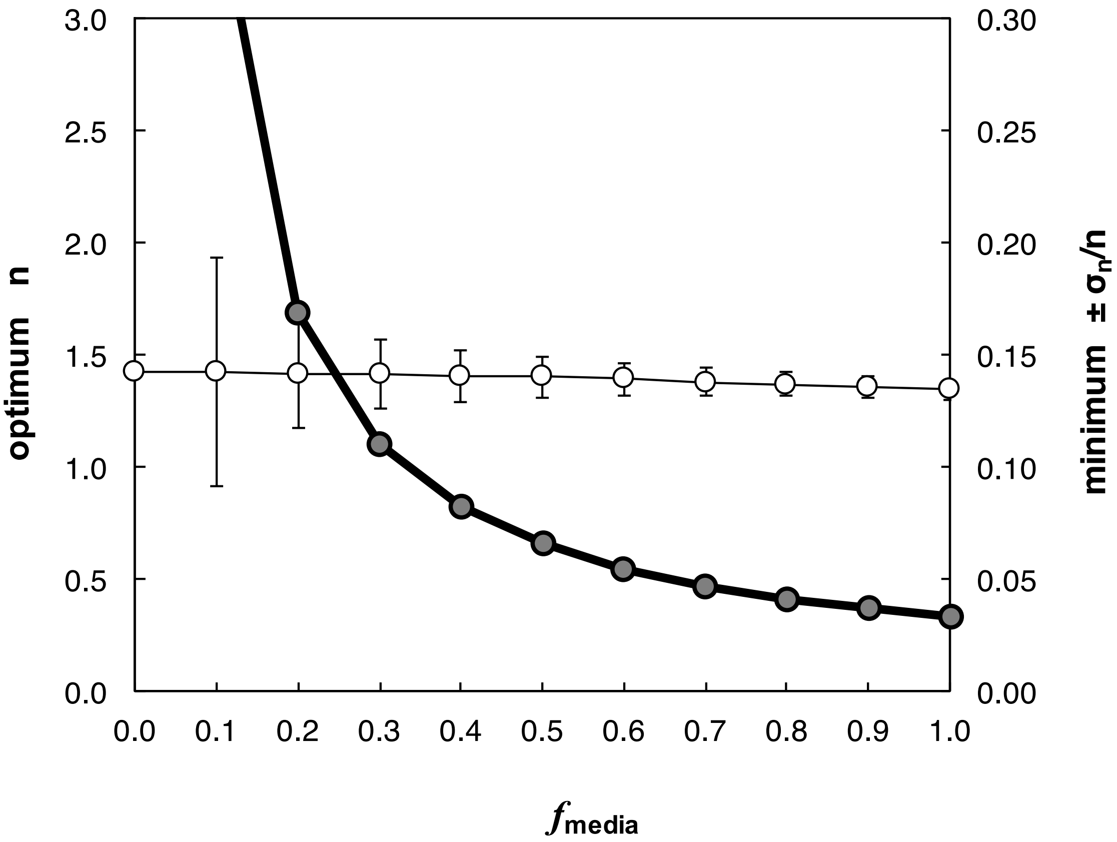

Figure S3. Estimated minimum relative uncertainties ($\pm\sigma_n/n$, filled circles, heavy line) and corresponding number of cell generations (n, open circles, thin line) as a function of f_{media} assuming $\alpha = 0.976 \pm 0.003$, $b_1 = -30.34 \pm 0.18$ cm^{-1}, $b_0 = 1157.04 \pm 0.05$ cm^{-1}, $f_o = 0.0110 \pm 0.0002$, and $\sigma_{\nu(C\text{-}C)} = 0.34$ cm^{-1}.

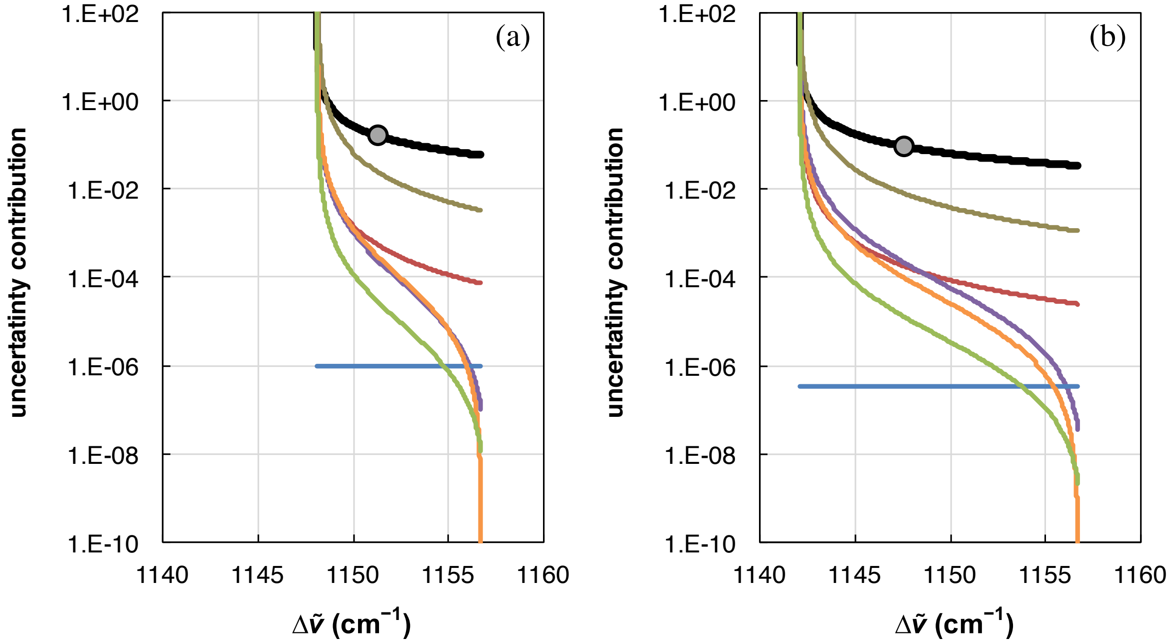

Figure S4. Modeled contributions of each measurement's uncertainty (f_o = blue, α = green, f_{media} = orange, b_1 = purple, b_0 = red, and $\langle \Delta\tilde{v} \rangle$ = brown) toward the total uncertainty ($\pm\sigma_n$; heavy black line) of the number of generations (n) determined via SCRR for f_{media} = (a) 0.3 and (b) 0.5, as a function of mean wavenumber. The uncertainty ($\pm\sigma_n$) for the optimum conditions (minimum $\pm\sigma_n/n$) to measure n are indicated by the filled circles. The contributions to the total uncertainty were calculated as the individual terms for each measurement under the radical of Eq. S25; i.e., as $(\partial n/\partial x)^2\sigma_x^{\ 2}$, where x = α, f_o, f_{media}, b_0, b_1, or $\langle \Delta\tilde{v} \rangle$.
